# Single-nucleus transcriptomics identifies cell cycle and synaptic pathway dysregulation during OPC-to-glioma progression

**DOI:** 10.3389/fncel.2026.1713437

**Published:** 2026-06-30

**Authors:** Dennis Huang, Angeliki Mela, Hye-Jin Park, Peter Canoll, Patrizia Casaccia

**Affiliations:** 1 Program in Molecular, Cellular and Developmental Biology at The Graduate Center of The City University of New, New York, NY, United States; 2 Neuroscience Initiative, Advance Science Research Center, Graduate Center of The City University of New York, New York, NY, United States; 3 Department of Pathology and Cell Biology, Columbia University Irving Medical Center, New York, NY, United States

**Keywords:** brain tumor, glia, neuron, p53, PDGF, progenitor, transcription

## Abstract

Gliomas are characterized by poor survival rate and limited options for treatment. Based on the transcriptional enrichment for oligodendrocyte progenitor cell (OPC) transcripts, the proneural glioma is thought to arise from transformation of OPCs. Here, we injected mutant BB-p53n OPCs (with *Trp53* deletion and PDGF-BB overexpression) into recipient mice and performed single-nucleus RNA sequencing (snRNA-seq) of the injected cells and of brain tissue at early and late time points after injection, coincident with neuroimaging detection of tumoral masses. Analysis of tumor-bearing brain samples, identified a cluster that was not detected in the normal brain, but was enriched for OPC markers (*Olig2),* cell cycle genes (*Myc*) and glioma markers (*Top2a, Sox2*), which we named “OPC-like.” The clusters with “OPC-like” signature, were also the ones with greater genomic distribution of inferred copy number variations (inferCNVs) and high proliferative rate, and were therefore denoted as “tumors.” The inferCNV genomic load was higher in late-stage samples compared to early ones, indicative of progressive genomic instability. Immunohistochemical analysis validated the high proliferative rate and widespread expression of the “OPC-like” markers TOP2A and SOX2. Pseudotime analysis of cycling cells identified a trajectory of decreasing cell cycle checkpoint regulation and increasing synaptic signaling from early to late timepoints. Thus, the early timepoints were characterized by the emergence of highly proliferative cell clusters with a unique “OPC-like” transcriptional signature and inferCNVs, and the late timepoints were characterized by further genomic spreading of inferCNVs, loss of cell cycle checkpoints and transcriptional changes consistent with increased neuron-glioma interactions

## Introduction

Gliomas are the most common and lethal type of primary brain tumors and are thought to originate from glial cells in the central nervous system (Komori, 2022; Louis et al., 2021; Zong et al., 2012). Despite advances in surgical techniques, radiotherapy, and chemotherapy, gliomas remain associated with poor prognosis and limited therapeutic options, particularly high-grade gliomas (Ostrom et al., 2015; Rajesh et al., 2017). Understanding the molecular and cellular mechanisms that drive glioma progression is crucial for the development of more effective diagnostic and treatment strategies. Oligodendrocyte progenitor cells (OPCs) have been suggested to be the cell of origin of proneural gliomas (Liu et al., 2011; Sonabend et al., 2013), and in the normal brain their proliferative state is highly responsive to mitogen concentration and neuronal activity (Bergles and Richardson, 2016). Since, in proneural gliomas, OPCs harbor several genetic alterations (Verhaak et al., 2010) we previously characterized the growth characteristics and the transcriptional and epigenetic differences between OPCs harboring *Trp53* deletion and PDGF-BB overexpression (BB-p53n OPCs) and those only carrying *Trp53* deletion (p53n) (Huang et al., 2024). We previously showed that the injection of mutant BB-p53n OPCs into the subcortical white matter of recipient mice resulted in the formation of gliomas, while the injection of p53n OPCs did not (Huang et al., 2024). However, the previous epigenetic and transcriptomic characterization of BB-p53n OPCs did not address the transcriptional changes occurring over time after the injection of the BB-p53n OPCs. We reasoned that a longitudinal snRNA-seq design of brain-samples collected at two time points after injection of the mutant BB-p53n as well as nuclei collected from cultured BB-p53n OPCs would allow us to identify the main events occurring in the brain between injection and detection of late stage tumors. Previous studies using snRNA-seq were used to characterize human glioblastomas (Neftel et al., 2019) and to characterize the importance of myeloid cells in murine glioma (Rajendran et al., 2023) or define the importance of glial progenitors in murine gliomagenesis (Weng et al., 2019). Our study follows this line of study, by adopting a longitudinal study design of early- to late-stage gliomas after the injection of OPCs with well defined genetic alterations. The results shown here are consistent with the emergence of highly proliferative populations of OPC, with a unique transcriptional signature characterized by positive regulation of the cell cycle and high expression of markers of the primitive OPC state. We also inferred CNVs from the datasets and noted a progressively wider genomic distribution from the early to the late collected brain samples, suggesting high genomic instability. Pseudotime analysis of the progression from early to late gliomas further identified the loss of regulatory checkpoints and the detection of an increased synaptic signaling transcriptional signature, consistent with the importance of neuron-glioma interactions driving tumor growth at the later stages, as previously reported (Venkatesh et al., 2015; Venkatesh et al., 2019).

## Materials and methods

### BB-p53n OPC *in vivo* injections

BB-p53n OPC isolation and infection were performed as described in Huang et al. (2024). In brief, primary mouse OPCs from either sex were isolated from the brain of *Trp53fl/fl* C57BL/6 (JAX:008462) pups at postnatal day 5–7 with a rat anti-mouse CD140a antibody (Fisher Cat# 558774), recognizing PDGFRα. OPCs were then transduced with a PDGFB-IRES-CRE retrovirus to obtain *Trp53*null and PDGFB overexpressing (BB-p53n) OPCs. Retrovirus production was performed as described in Lei et al. (2011). Cell implantation was performed, according to IACUC approved protocols, by stereotactic intracranial injection of 50,000 OPCs in 1 μL of SATO media, at a flow rate of 0.25 μL/min with a Hamilton syringe, as previously described (Upadhyayula et al., 2023). C57BL/6 J Mice (JAX:000664) were anesthetized by intraperitoneal injection with Ketamine/Xylazine (100 mg/kg and 10 mg/kg, respectively) and assessed for lack of reflexes by toe pinch. Cells were injected through a burr hole that was made with a 17-gauge needle, 2 mm lateral and 2 mm anterior to the bregma, 2 mm deep into the brain parenchyma aiming for subcortical white matter.

### Tumor detection and tissue collection

The same batch of BB-p53n OPCs was injected into the brain of six syngeneic female mice, as described above. Mice were assessed daily for clinical signs, while tumor growth was assessed by MRI monitoring using a Bruker BioSpec 9.4 Tesla Small Animal MR Imager (CUIMC Oncology Precision Therapeutics and Imaging Core, OPTIC). Tissue was collected from three mice with detection of early-stage tumors at 45dpi (E1, E2, and E3), and three mice with late-stage tumors at 60dpi (L1), 73dpi (L2) and 64dpi (L3) and contained the tumor as well as normal brain tissue. Mice were euthanized by cervical dislocation and the brains were rapidly removed to preserve the transcriptional signatures. The right frontal lobe tissue in the injected hemisphere 2 mm anterior to bregma, containing the tumor as well as normal-appearing parenchyma, was immediately flash frozen in liquid nitrogen and stored at -80 °C, while the rest of the tissue was placed in 4% paraformaldehyde (PFA) to be fixed for histological analysis. After 24 h of tissue fixation, the hemisected brain saved for histology was washed, kept in PBS and sent to the Molecular Pathology Shared Resource (CUIMC MPSR) for paraffin embedding, sectioning and histological staining, such as Hematoxylin–Eosin. The remaining sections were used for immunohistochemistry using different antibodies.

### Single nucleus isolation, library preparation and initial processing

Before dissociation, the frozen samples underwent additional trimming in order to generate similar size tissue blocks, centered around the coordinates of the injection site and containing both the tumor and normal brain tissue. Trimming was performed by thawing the samples in ice cold Miltenyi tissue storage solution (cat# 130–100–008). Subsequent steps to extract nuclei followed a slightly modified version of the 10X nuclei isolation kit (cat# 1000493) protocol for gene expression libraries. The yield of cell nuclei varied across samples, with greater yield obtained from the early tissue samples (E1: 930,000; E2:530,000; E3:890,000) and lower yield for the late tissue samples (L1:530,000; L2:280,000; L3: 450,000), possibly resulting from differences in overall tissue damage due to tumor growth and necrosis. After debris removal with Milteny’s solution (cat# 130–109-398), the nuclei were resuspended in in PBS containing 0.1% BSA and then isolated according to the protocol of the 10X nuclei isolation kit. After extraction, the purified nuclei were resuspended at a concentration of 10^6^/mL and subjected to 10× 3’ Gene Expression library preparation (Chromium Next GEM Single Cell 3′ Kit v3.1, 16 rxns PN-1000268). All the longitudinally collected samples were sequenced at the same time, with a target of 10,000 nuclei per sample and 40,000 reads per nucleus, using a Novaseq 6,000 sequencer. Samples from cells were processed separately. A plot of the genes per nucleus for each dataset can be found in Supplementary Figure 1. All data deposited in GEO, accession number GSE309333.

### snRNA-seq raw data processing, integration, clustering analysis

Raw sequencing data were put through the default 10X cellranger (Cellranger v8.0.1) pipeline for alignment and gene annotation using mm39 reference files provided by 10X. Nuclei were first filtered using the Cellranger’s barcode calling algorithm (with the expect-cells set to 15,000), followed by additional manual filtering by total number of detected genes. The cutoffs were defined to remove far outliers and maintain the primary population of cells in each sample with +/−1.5 times interquartile range, as reflected in Supplementary Figure 1. The data on the BB-p53n OPCs were obtained from cultured cells, rather than tissue samples and therefore were characterized by an overall higher distribution of genes per cell and needed a different cutoff (parameters set as follows: 600 < = nGenes <= 6,000 for Early, Late and normal Brain; 2000 < = nGenes <= 8,000 for BB-p53n OPCs). A detailed description can be found in Supplementary Table 1. Filtered nuclei were imported into R for further analysis. Normalization, dimensional reduction, unsupervised clustering, automated annotation, module scoring (enrichment analysis), and most visualizations of single nucleus data were all done through the Seurat package (v5.1.0) (Hao et al., 2021; Stuart et al., 2019; Butler et al., 2018; Satija et al., 2015). Three early-stage and three late-stage tumor samples were merged into a single Seurat object and run through the normal snRNA-seq Seurat pipeline: normalizing, finding variable genes, scaling data and calculating principal components (PCs). Each data set was normalized with log normalization (the scale factor set to 1,000) and number of variable genes set to 3,000. After normalization, the data were scaled and adjusted by linear regression according to total counts per cell. The significant PCs (1:20) were used to generate Uniform Manifold Approximation and Projection (UMAP) plots and clusters in the data (resolution set to 1.0 and number of neighbors set to 200). 10X normal mouse brain dataset was processed the same way as tumor datasets. Finally, Seurat’s RPCA integration method was implemented to reduce batch related variation. All UMAP and violin plots were created using Seurat package tools.

### Single-nucleus RNA sequencing annotation

Azimuth was used to rapidly annotate the early- and late-stage tumor datasets described above to their closest CNS cell type (Yao et al., 2021). Annotations of multiple types of neurons were combined into either GABAergic (Lamp5, Meis2, Pvalb, Sncg, Sst, Sst Chodl, Vip) or Glutamatergic Neurons (L2/3 IT, L5 ET, L5 IT, L5/6 NP, L6 CT, L6 IT, L6 Car3, L6b). Nuclei with Azimuth prediction and mapping scores lower than 0.75 were further assessed by Seurat’s module scoring, calculating levels of enrichment by gene sets. Nuclei with low Azimuth prediction and mapping scores that also received a OPC module score greater or equal to the median were manually annotated as OPC-like. OPC cell type gene sets were obtained from McKenzie et al. (2018).

### Differential expression and enrichment analysis

Pseudobulk differential expression analysis was performed by analyzing nuclei from each of the three mice with early-stage tumors and three with late-stage tumors and calculating the summation of counts per gene. After summation, the six subsets were treated as 3 bulk RNA-seq for each set of early- and late-stage tumor bearing samples and used for differential expression analysis. In the case of the comparison between “OPC-like vs other cell types”, each of the six samples obtained from mice (three early- and three late-stage tumor bearing sample datasets) had their nuclei divided into OPC-like nuclei and all other cell types. This resulted in six observations for OPC-like nuclei and six observations for other cell types separated by sample/mouse. In the case of “late versus early tumor cells”, the identified tumor nuclei were separated by mouse resulting in three observations of late-stage and three for early-stage tumor-bearing samples. Once pseudobulk count matrices were prepared, the standard differential expression analysis procedure was followed as instructed by the edgeR Bioconductor package, including normalization (calcNormFactor()), count transformation (voom()), and linear modelling (lmfit()) (v4.0.16) (Robinson et al., 2010).

Gene set enrichment analysis (GSEA) was performed with the clusterProfiler, using the gseGO(), GSEA() functions (v4.10.0) package and Org. Mm.eg.db (v3.18.0) annotation database (Subramanian et al., 2005; Yu et al., 2012). Gene list inputs for GSEA were filtered by adjusted *p*-value less than 0.01, calculated by differential expression analysis. CNS cell type gene sets were obtained from McKenzie et al. (2018). Curated cell cycle progression gene sets were obtained from Tirosh et al. (2016), Neftel Signatures were obtained from Neftel et al. (2019), the OPC-like signature was defined as the top 50 genes with adjusted *p* value *<=0.005* and log fold change greater than 1.0, and other gene sets obtained from the Gene Ontology database (Ashburner et al., 2000). All enrichment dotplots were generated with the enrichplot (v1.22.0) package (Yu, 2024). All volcano plots were generated using ggplot2 (v3.5.1) (Wickham, 2016).

### Inferred CNV analysis

Nuclei from the merged Seurat object containing normal mouse brain, early samples, and late samples (~80,000 nuclei), were randomly divided into 4 equal subsets - using R – to be analyzed using the inferCNV package (v3.20) (inferCNV, 2025), in order to overcome limited memory in local hardware. In order to cover the entire population of ~80,000 nuclei, inferCNV was iterated for 4 times, each time choosing ~20,000 nuclei that were not included in the previous draw. 5,000 cells from a normal mouse brain dataset provided by 10X Genomics were used as reference. The raw count data from each subset were used as input for inferCNV, for each of the four draws, using the suggested parameters for 10X data (cutoff = 0.1, denoise = TRUE, HMM = TRUE, cluster_by_groups = TRUE). To visualize data from inferCNV analysis in UMAPs, the output from inferCNV was joined with either early or late metadata by matching nuclei barcodes. Scaled proportions of CNV within each chromosome were added together for a “infer CNV score” for each nucleus across their genome.

### Tumor cluster identification

Early- and late-stage datasets were integrated with 5,000 cells from a normal mouse brain dataset provided by 10X Genomics which was prepared with the same single nucleus gene expression kit as the tumor data. Integration was performed as described above with Seurat’s RPCA method. Those clusters whose transcriptional signature of the nuclei was not identified in the normal brain were identified as “OPC-like,” as their signature included oligodendrocyte markers, such as *Olig2* and glioma markers, such as *Top2a*. All the transcripts that were upregulated greater than 1 FC in the “OPC like” clusters and with an adjusted *p* value *<=0.005* were considered as defining the “OPC-like signature” and the top 50 genes were used to generate a UMAP of enrichment. Similarly, the top 50 genes for the OPC and for the NPC Neftel’s signature (Neftel et al., 2019) were used to identify the clusters enriched for such a signature. Clusters that were enriched for the “OPC-like” and for the “NPC and OPC” Neftel’s signatures were detected only in datasets which integrated the normal brain with the early- and late-stage tumor bearing samples and were characterized by the presence “tumor” clusters. Further validation resulted from the identificat of inferred CNV enrichment, across the genome. Since CNV can also spontaneously occur in normal tissue (Erickson et al., 2022), but it is usually limited to smaller regions, less than 1 Mb (Rohrback et al., 2018), the “infer CNV score” described above was used to characterize cluster distribution and enrichment of CNVs across the data. The same clusters enriched for the “OPC-like” and “OPC and NPC Neftel’s” signature, were also the ones enriched for inferred CNVs. These “tumor” clusters were filtered out from the early- and late-stage tumor bearing sample datasets and processed for further downstream analysis (e.g., differential expression, GSEA).

### Cell cycle position analysis with TRICYCLE

Tumor cluster nuclei were filtered, normalized as described above and analyzed for cell cycle positions with TRICYCLE (Zheng et al., 2022). Analysis was done using the project_cycle_space() and estimate_cycle_position() functions under default parameters. 0*π* and 2π positions are highly associated with cells in G1/G0. Between 0.5π and π is S phase. Between π and 1.5π is G2/M phase. Nuclei were labelled as coming from cycling cells if their tricycle position was between 0.5π and 1.5π, and from quiescent cells if outside that range. Cell cycle plots (density plot) were made following TRICYCLE’s instruction with ggplot2 tools (v3.5.1).

### Pseudotime analysis (monocle3)

Cycling and quiescent tumor nuclei were filtered out of both early- and late-stage datasets according to methods described in previous sections. As one Seurat object, the tumor nuclei were processed and re-clustered by the Seurat package. The data were then input to monocle3 (Trapnell et al., 2014) tools to calculate pseudotime values. Pseudotime was calculated using the learn_graph() function under default parameters and roots were set to the cluster with the highest normal OPC enrichment signature, since the biological question addressed in this paper addresses how the injection of mutant BB-p53n OPCs evolve into early- and late- stage tumors. Pearson’s correlation analysis between pseudotime and gene expression was performed using the cor() function in base R. Nuclei barcodes were aligned with their respective pseudotime value and gene expression data. Correlation was calculated for each vector of expression values per gene and pseudotime values. Positive correlation values designated a higher likelihood that gene expression increased as pseudotime increased and vice versa for negative correlation values. We used this analysis for cycling tumor cells only, quiescent tumor cells only and a combination of cycling and quiescent tumor cells. Pseudotime specific UMAP and temporal gene expression figures were plotted using the Seurat’s dimplot() and monocle3’s plot_genes_in_pseudotime() function, respectively.

### Immunohistochemistry

Paraffin-embedded 5 μm tissue slices were processed for immunohistochemistry. Briefly, following deparaffinization with xylene and ethanol, sections were boiled in Sodium Citrate buffer, pH 6.0, for 10 min at high pressure in a pressure cooker. After cooling down, sections were treated with 1% hydrogen peroxide to quench endogenous peroxidases, then washed in phosphate buffer saline (PBS) and incubated in blocking solution (0.5% Triton-X in PBS with 5% Horse Serum), at room temperature for 30–60 min. Following overnight incubation with primary antibody in blocking solution at 4 °C, sections were washed in PBS and incubated with biotinylated secondary antibody for 1 h and then with the ABC reagent for 30–45 min, according to the Vectastain Elite ABC kit instructions (Vector Laboratories). DAB (Dako) incubation was used to visualize staining. Sections were then dehydrated and mounted with Permount. Primary antibodies for TOP2a (Abcam ab52934, rabbit 1:200) and SOX2 (Millipore AB5603, rabbit 1:200) were used.

### Glioma cell line

The BB-p53n glioma cell line was derived as previously described in Lei et al., (2011). Once female mice showed evidence of tumor morbidity, their brains were harvested after proper euthanasia. Tumors were then resected, dissociated and cultured in defined tissue culture media containing high glucose DMEM (5.4 g/L D-glucose, L-glutamine), 0.5% FBS, N2 supplement, PDGF-AA (10 ng/mL), bFGF (10 ng/mL), and antibiotic–antimycotic solution. In subsequent experiments, BB-p53n OPCs and glioma cells were cultured under the same conditions in SATO media.

### Edu incorporation

Cells were seeded in 8-well chamber slides (Thermo Fisher Scientific, 154941PK) and incubated overnight. EdU incorporation was performed using the Click-iT™ Plus EdU cell Proliferation Kit (Thermo Fisher Scientific, C10637) following the manufacturer’s instructions. Cells were treated with 10 μM EdU for 2 h, washed with PBS, and fixed with 4% paraformaldehyde (PFA) for 15 min at room temperature, followed by 2 PBS washes. Fixed cells were then processed for immunocytochemistry. Briefly, cells were permeabilized with 0.2% Triton X-100 in blocking buffer (PGBA containing 5% goat serum) for 1 h at room temperature and incubated with primary antibody at 4 °C overnight. After 3 PBS washes, cells were incubated with appropriate secondary antibody for 1 h at room temperature and washed 3 times with PBS. EdU detection was performed by staining with Alexa flour 488-conjugated azide for 30 min at room temperature in the dark. Cells were then washed, counterstained with DAPI, and mounted using Fluormount G mounting medium (Thermo Fisher Scientific). Confocal images were acquired using the Zeiss LSM-800 fluorescent microscope and Zen Blue software. For quantification of EdU-positive cells, at least 3 random fields per well were imaged, and the percentage of EdU-positive cells was calculated for each field.

### Immunocytochemistry

Cells for immunocytochemistry were seeded in 8-well chamber slides (Thermo Fisher Scientific, 154941PK) and fixed with 4% paraformaldehyde (PFA) for 15 min at room temperature. Fixed cells were permeabilized and blocked in PGBA (Phosphate buffer with 0.1% gelatin, 1%BSA, 0.002% Sodium Azide) containing 0.2% (vol/vol) Triton X-100 (Fisher Scientific cat#AAA16046AP) and 5% normal goat serum (Vector Laboratories cat#S-1000-20) at room temperature for 1 h. Primary antibodies were applied overnight at 4 °C or 1 h at room temperature followed by incubation of appropriate secondary antibodies conjugated with fluorophores. DAPI (life technologies Cat#D21490) in PBS is applied for 10 min and washed before mounting. Confocal images were captured using the Zeiss LSM-800 fluorescent microscope and Zen Blue software. Blinded quantification of the immunofluorescent intensity was performed using Fiji/ImageJ (Schindelin et al., 2012). Bar plots generated in Graphpad Prism (Graphpad version 10.0.0 for Windows). Antibodies used are listed as follow: anti-CD140a (PDGFRα, 1: 400, BD Biosciences #558774), anti-OLIG2 (1:500, Millipore #MABN50), anti-Topoisomerase alpha (1:1000, Abcam #ab52934), anti-SOX2 (1:1000, Cell Signaling #23064S), goat anti-rabbit IgG Alexa Fluro 488 (1:500, Invitrogen #A-11008), goat anti-mouse IgG Alexa Fluro 555 (1:500, Invitrogen #A-21422), goat anti-rat IgG Alexa Fluro 647 (1:500, Invitrogen #A-21247).

## Results

### Single-nucleus RNA-seq of mutant BB-p53n OPCs identifies a population of progenitor cells resembling the primitive OPC phenotype

**Figure1:**
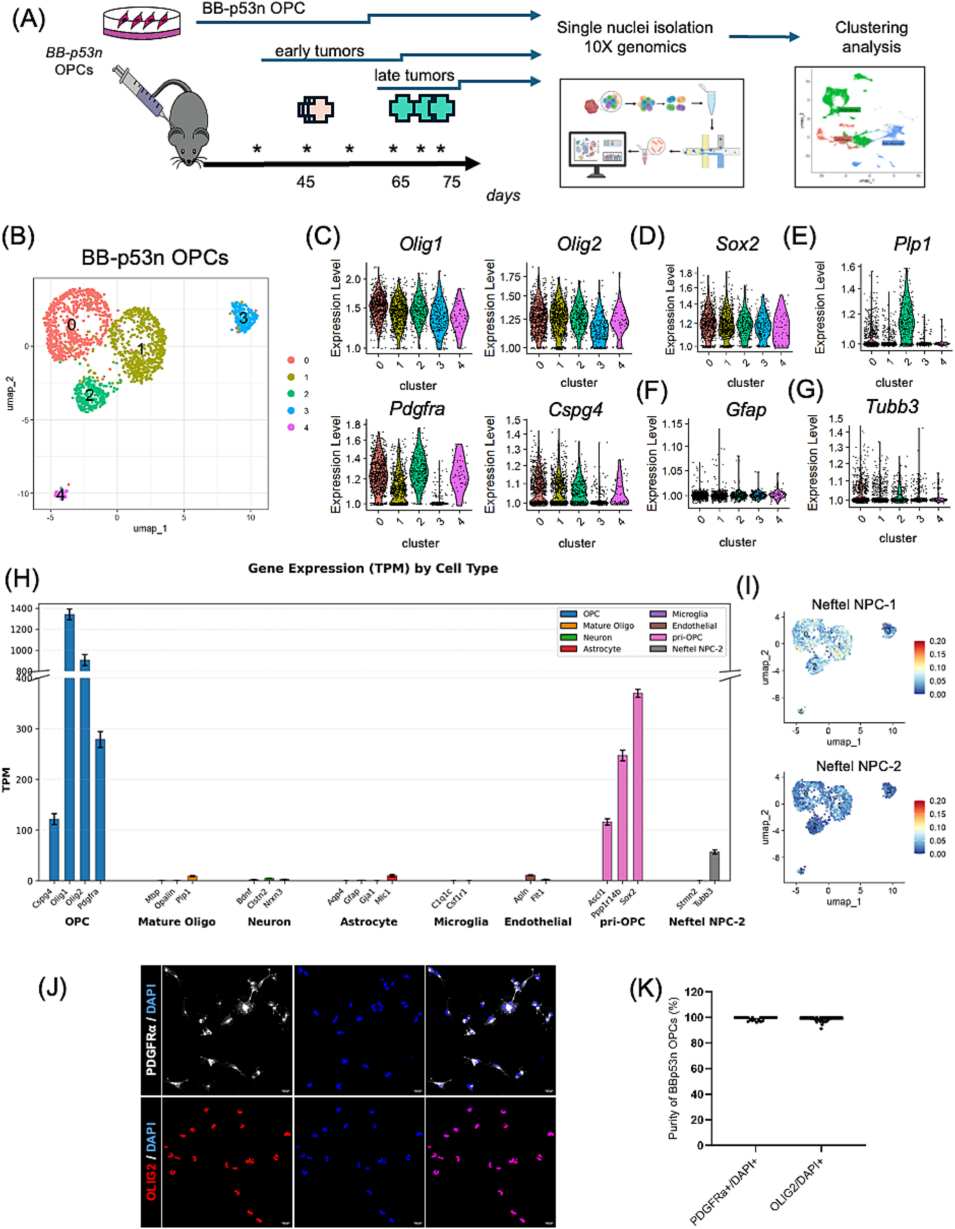
BB-p53n OPCs are transcriptionally heterogeneous. **(A)** Experimental design of the single nucleus transcriptomic study of early and late gliomas induced by injection of mutant (BB-p53n) OPCs into recipient mice. **(B)** UMAP plot of snRNA-seq from cultured BB-p53n OPCs. **(C–G)** Violin plots representing transcript levels in each cluster annotated in the UMAP plot in panel B: OPC markers *Olig1Olig2, Pdgfra, Cspg4* **(C)**, stem cell marker *Sox2* **(D)**, oligodendrocyte marker *Plp* **(E)**, astrocyte marker *Gfap* **(F)**, neural progenitor marker *Tubb3* **(G)**. **(H)** Barplot showing the TPM values of BB-p53n OPC bulk RNA-seq data from Huang et al., 2024 for the transcripts specific for the distinct cell types indicated on the X axis. **(I)** UMAP plots of BB-p53n OPCs. The color of the dots represents the enrichment levels of the transcriptional signature identifying Neftel NPC-1 and NPC-2 cells, with blue representing less enrichment and red representing higher enrichment. **(J)** Representative confocal images of cultured BB-p53n OPCs stained for PDGFRa (white), OLIG2 (red) and DAPI (blue) as a nuclear counterstain. Scale bar = 20 μm. **(K)** Scatterplot of data quantified from images similar to the one shown in panel **J**. Each point represents the calculated percentage of PDGFRa+/DAPI+ cells or OLIG2+/DAPI+ cells per image field. Six to 12 image fields were quantified from 3 distinct experiments.

We had previously characterized the transcriptional and epigenetic differences between mutant BB-p53n OPCs, which generated tumors when injected into the brains of syngeneic mice, and p53n OPCs, which did not form tumors. However, the steps occurring in the normal brain after the injection of mutant BB-p53n OPCs and leading to the formation of early-stage and then late-stage gliomas remained undetermined. To better understand this process, we reasoned that it would be important to start by providing a thorough molecular characterization of the cultured mutant BB-p53n OPCs using snRNA-seq (Figure 1A). UMAP representation of the BB-p53n OPC data identified them as a heterogeneous population composed of five clusters (Figure 1B). While all of the five clusters were characterized by the expression of the oligodendrocyte lineage-specific transcription factors *Olig1* and *Olig2* (Figure 1C; Supplementary Figure 2A), and to a certain degree also of *Sox2* (Figure 1D; Supplementary Figure 2D), cluster 2 was characterized by the expression of *Plp1* (Figure 1E; Supplementary Figure 2C) and cluster 3 by lower levels of the OPC markers *Pdgfra* and *Cspg4* (Figure 1C; Supplementary Figure 2B). None of the identified clusters showed enrichment for cell-type-specific transcripts enriched in neuronal, astrocytic (Figure 1F; Supplementary Figure 2E) or neural progenitor cells (Figure 1G; Supplementary Figure 2F). Bulk RNA-seq analysis of the BB-p53n OPCs, using data from our previous study (Huang et al., 2024), further validated the enrichment of genes specific for OPCs and primitive OPCs (Figure 1H). The negligible levels of *Tubb3*, a gene previously reported as one of the transcripts expressed in neural progenitor cells, was consistent with the lack of enrichment for Neftel NPC-1 and NPC-2 signatures (Neftel et al., 2019; Figure 1I). As the gene signature of any cell type consists of more than a single gene, we mapped the five clusters with the signature of distinct cell types based on previous publications (McKenzie et al., 2018), which revealed an enrichment for the OPC and primitive OPC signature, especially in cluster 0, cluster 1 and cluster 2 (Supplementary Figure 3). At a cellular level, the mutant OPC cultures were characterized by the expression of PDGFRα and OLIG2 proteins and by the absence of any contaminating cell type (Figures 1J,K). Overall, these data suggest that the mutant BB-p53n OPCs were characterized by the presence of a signature consistent with a primitive OPC phenotype, previously reported to be involved in gliomagenesis (Weng et al., 2019).

### Single nucleus RNA-seq of brain samples from mice with early- and late-stage gliomas reveals the emergence of an expanding population of OPC-like cells characterized by the expression of glioma markers

**Figure 2:**
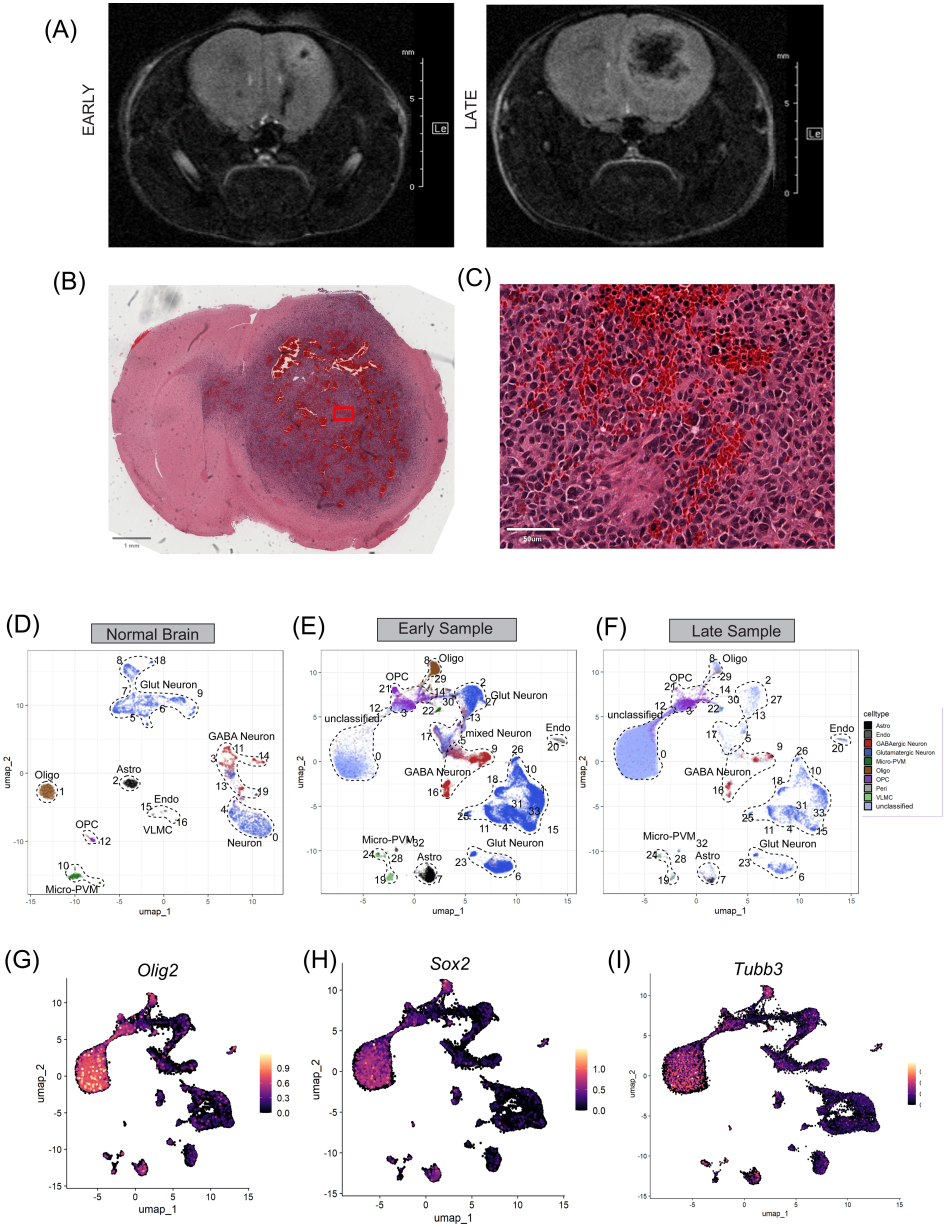
BB-p53n OPCs injected in the subcortical white matter of syngeneic mice, form gliomas characterized by the presence of cells with a unique “OPC-like” transcriptional signature. **(A)** MRI coronal images of the head of mice at time of harvesting for early- and late-stage tumors. The images are representative of one of three early and three late mice. Scale bar in millimeters. **(B,C)** Representative H&E staining of late-stage tumor at low **(B)** and high **(C)** magnification. Scale bars represent 1 mm **(B)** and 50 μm **(C)** respectively. The image is representative of the histopathology of late-stage tumors, with all the features of pseudo-palisading necrosis and extensive vascularization. **(D–F)** UMAP plots of dimensionally reduced annotated single nucleus data **(D)** of normal mouse brain (5,000 nuclei) from the 10X database and **(E)** of nuclei from early (38,863 nuclei) and **(F)** late (44,948 nuclei) tumor-bearing brain tissue samples. Automatic annotation of CNS cell types includes astrocytes (ASTRO), endothelial cells (ENDO), glutamatergic neurons (GLUT), GABAergic neurons (GABA), oligodendrocytes (Oligo), OPCs, microglia (Micro), pericytes (Peri), and vascular leptomeningeal cells (VLMC). Note the emergence of a newly identified cluster, which we annotated as “unclassified” in the early and late samples, as it was not found in the normal brain set and did not match the “OPC” or “oligodendrocyte” classification. **(G–I)** UMAP plots showing the enrichment of **(G)** *Olig2*, the stem cell marker *Sox2* **(H)** and the neural progenitor marker *Tubb3* **(I)**, in the “unclassified” cluster.

To address the mechanisms of glioma formation and progression, we injected 50,000 mutant BB-p53n OPCs into the subcortical white matter of female recipient mice with the same genetic background and monitored them for the presence of clinical signs and for imaging evidence of tumor growth by conducting weekly MRI imaging. Mice were sacrificed at two subsequent time points after the mutant OPC injection, which we arbitrarily defined as two “stages,” although the processed brain tissue blocks contained tumor as well as normal brain tissue (Figure 1A). The “early-stage” collection time points were defined by the MRI detection of small masses, typically occurring around 45 days post-injection (dpi). The “late-stage” collection time points were defined by the MRI detection of large tumors and progressively deteriorating clinical signs, typically occurring between 60 and 73 dpi (Figure 2A). For this reason, we referred to them as “early-stage” and “late-stage” tumor-bearing brain tissue samples. Three mice were sacrificed at each early and late time point. The hemisected brain containing the injection site was divided into two halves: the anterior portion surrounding the injection site coordinates was flash-frozen in liquid nitrogen and processed for nuclear extraction while the posterior portion was processed for histological validation of the tumors. Besides clinical and radiological evidence, the presence of tumors was confirmed by the detection of the characteristic histological hallmarks of gliomas, including neovascularization and pseudo-palisading necrosis (Figures 2B,C).

Trimmed brain tissue blocks, containing tumor as well as normal-appearing tissue, were further processed for nuclear extraction and subsequent 10× 3’ Gene Expression sequencing. Raw sequencing data of the harvested early- and late-stage brain samples along with a 10X provided normal mouse brain dataset were processed through the default 10X cell ranger pipeline for alignment and gene counts. After dimensional reduction and cell type annotation by Azimuth, in both early- and late-stage tumor-bearing samples, we detected a cluster which did not match any of the identifiable normal brain cell types (Figures 2D–F). Azimuth could not confidently assign the transcriptional signature to normal OPCs, and therefore this cluster remained unclassified in the tumor-bearing samples (Figures 2E,F). When we considered the differential transcripts in this cluster as compared to the rest of the cell type, with a cut-off of adjusted *p* value *<0.005* and FC > 1, we noted the presence of several genes related to cell division (Supplementary Table 2). The UMAP distribution revealed high levels of the *Olig2* transcript (Figure 2G), *Sox2* (Figure 2H), and *Tubb3* (Figure 2I), along with *Top2a* (Supplementary Figure 4A) and *Myc* (Supplementary Figure 4B) in this “unclassified” cluster in the tumor-bearing samples. Since this cluster was not detected in the normal brain dataset (Supplementary Figures 4C,D), and was characterized by the expression of *Olig2* and *Sox2,* we named it “OPC-like.” Of note, the early-stage samples (Figure 2E; Supplementary Figure 4E) were characterized by cell clusters with abundant representation of GABAergic, glutamatergic, mixed neurons, astrocytes, endothelial and other cell types, which were consistent with a good preservation of the parenchyma. In contrast, the late-stage tumor-bearing tissue samples were characterized by the progressive enlargement of the OPC-like cluster (Supplementary Figures 4E,F) and by sparse clusters of glutamatergic, GABAergic neurons and other cell types (Figure 2F), consistent with both the MRI imaging of large lesions (Figure 2A) and with the extent of necrosis and tissue damage detected by histopathology (Figures 2B,C).

**Figure 3:**
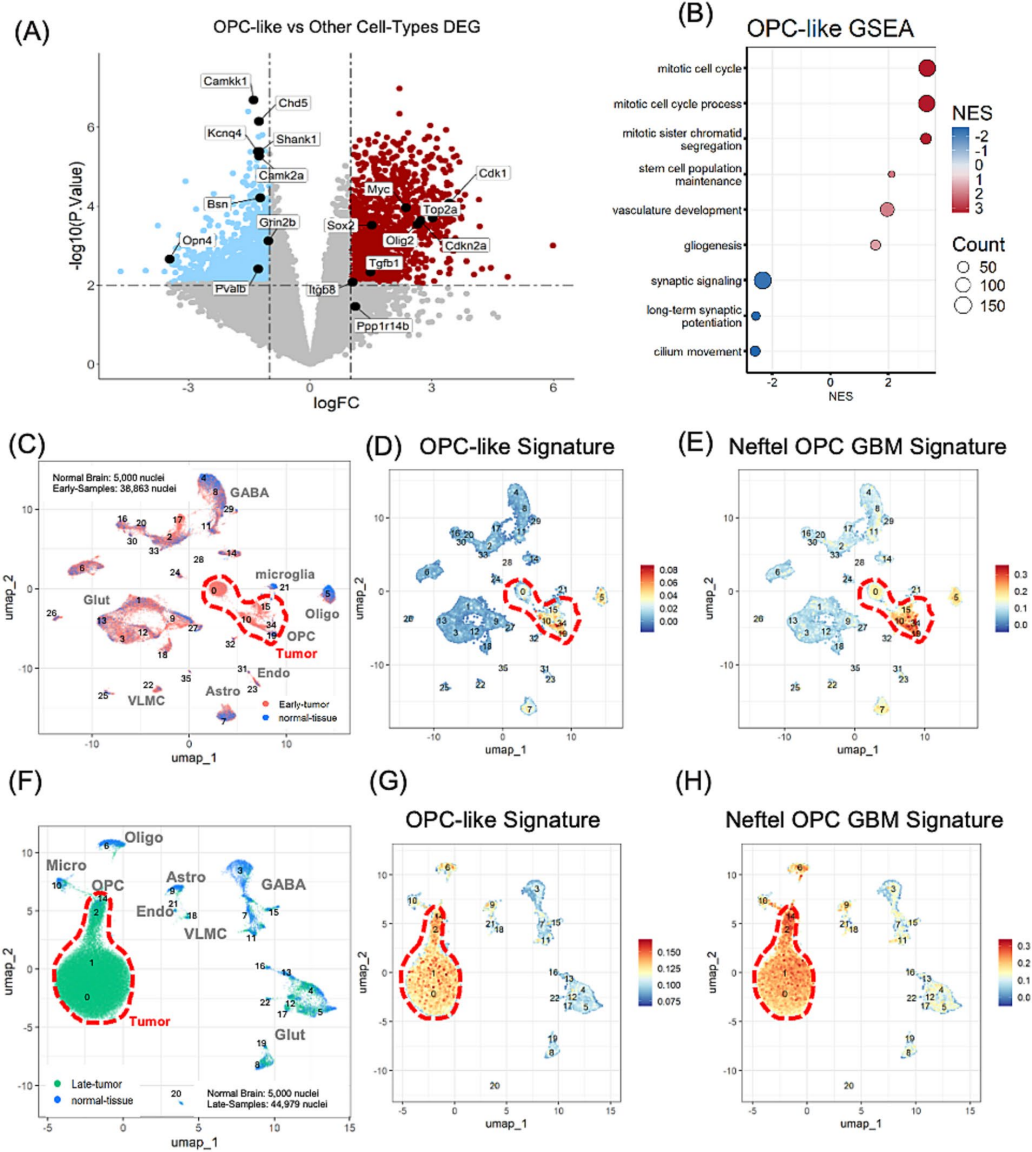
Characterization of the “OPC-like” signature and identification of “tumor” clusters. **(A)** Volcano plot of the differentially expressed genes between the “OPC-like” cluster and all the other clusters, resulting from the pseudobulk analysis of the six datasets from tumor-bearing datasets (3 early and 3 late brain samples). Only transcripts with adjusted *p* value <= 0.05 and FC > 1 are colored. Please refer also to Supplementary Table 2 for extended gene list. **(B)** Gene set enrichment analysis of genes differentially expressed in the “OPC-like” cluster compared to all the other cell types. Y-axis: gene ontologies, X-axis: normalized enrichment score (NES). Dot size refers to gene counts and color refers to adjusted *p*-value, with red being the most significant. Values to the left of the 0 represent downregulated pathways and those to the right represents upregulated pathways. Please refer to Supplementary Table 3 for more details. **(C)** UMAP plot of the integrated normal brain and early-stage tumor-bearing brain sample, identifying 35 clusters. **(D,E)** Feature plots, showing the cluster enrichment for cells with an “OPC-like” **(D)** or a Neftel OPC glioblastoma signature **(E)** in the early-stage tumor-bearing samples. **(F)** UMAP plot of the integrated normal brain and late-stage tumor-bearing brain sample, identifying 22 clusters. **(G, H)** Feature plots, showing the cluster enrichment for cells with an “OPC-like” **(G)** or a Neftel OPC glioblastoma signature **(H)** in the late-stage tumor-bearing samples.

To address the issue of reproducibility, we then conducted a pseudo-bulk analysis of the same data, but using results from individual mice and compared the “OPC-like” signature in the three early- and three late-stage tumor-bearing samples, in order to identify genes differentially expressed in this cluster compared to the rest of the cells. The volcano plot analysis revealed an enrichment for genes related to cell division, such as *Myc*, *Cdk1*, *Top2a* which were also previously reported in gliomas (Cencioni et al., 2023; Zhou et al., 2018; Weng et al., 2019), while genes related to synaptic signaling, such as *Shank1, Camk1, Pvalb* were down-regulated (Figure 3A). Consistently, gene set enrichment analysis (GSEA) of the differentially expressed genes in the “OPC-like” cluster, compared to all the remaining nuclei, revealed an enrichment in gene ontologies related to cell division, vasculature development and gliogenesis, while categories related to synaptic transmission were downregulated (Figure 3B). Thus, the “OPC-like” cluster was characterized by a transcriptional signature that was not the one of normal OPCs or neurons, but rather enriched for primitive OPC markers and proliferation genes (Supplementary Table 3). This cluster was not identified in normal brain datasets, as it was found only in the analysis containing datasets from the tumor-bearing brain tissue samples.

### Identification of tumor cell clusters

**Figure 4:**
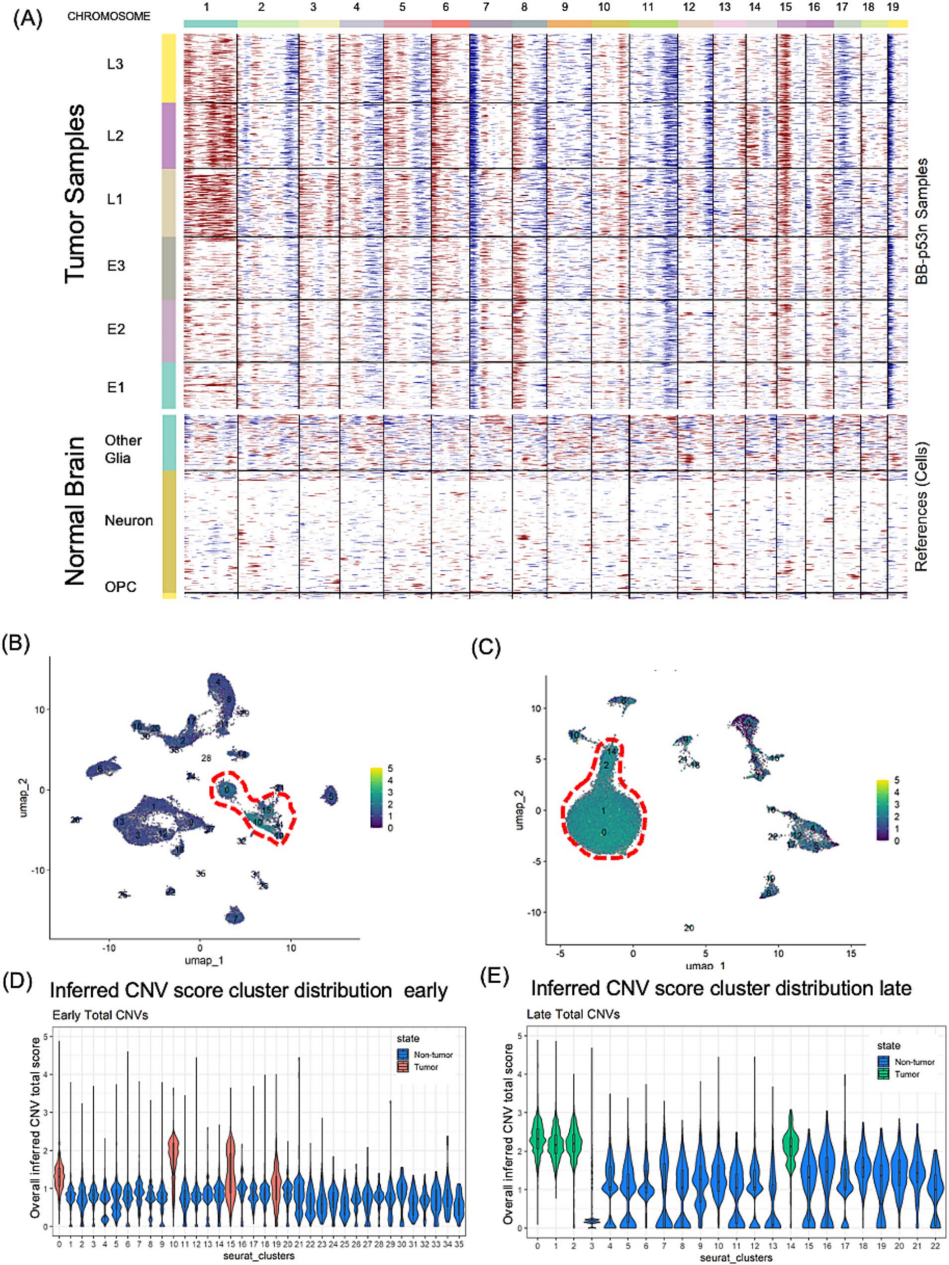
Widespread chromosomal distribution and cluster enrichment of inferred CNVs in the early- and late-stage tumor-bearing samples. **(A)** Genome-wide distribution of CNVs inferred from 20,000 randomly selected nuclei drawn from the three early- (E1–E3) and three late- (L1–L3) stage tumor-bearing tissue samples. The normal brain was used as reference and subcategorized into OPCs, neurons, and other glia. Chromosomes are organized in columns, each identified by a color and a number. Red color indicates inferred amplifications and duplications, and blue color indicates inferred losses or deletions. **(B,C)** UMAP plots representing clusters characterized by infer CNV score described in methods in early **(B)** and late **(C)** samples. Green/yellow colors represent enrichment. **(D,E)** Cluster distribution of inferred CNVs. The *X*-axis identifies the clusters detected in the early **(D)** and late **(E)** stage samples, while the *Y*-axis represents the infer CNV score. Violin plots in boxplots represent quantile ranges of the data.

We then integrated the datasets from either the early- (Figures 3C–E) or late- (Figures 3F–H) stage tumor-bearing brain tissue samples with the 10X normal mouse brain dataset, and identified 35 clusters in the early-stage (Figure 3C) and 22 in the late-stage samples (Figure 3F). When we visualized the UMAP distribution of the “OPC-like signature” in the integrated datasets between normal brain and early tumor-bearing sample, we noted five enriched clusters (Figure 3D), which were enriched for the Neftel’s OPC glioblastoma signature (Figure 3E). Similarly, in the late-stage tumor-bearing samples, we detected enrichment for the “OPC-like” signature (Figure 3G) in the same clusters that were also enriched for the Neftel’s OPC glioblastoma signature (Figure 3H). Those “tumor clusters” showed also an enrichment for the Neftel’s NPC glioblastoma (Neftel et al., 2019) signature (Supplementary Figure 5). As additional step to identify these clusters as tumoral, we performed an inferred CNV analysis (Figure 4A; Supplementary Figure 6), using the inferCNV package (inferCNV, 2025). A genome-wide representation of inferred CNVs revealed a progressive increase of inferred CNVs from early- to late-stage tumor bearing samples. The early-stage tumor bearing samples, for instance, showed a large number of duplications and amplifications on chromosomes 1, 7, 8, 15 and 16 and deletions on chromosomes 7, 11, 17 and 19 (Figure 4A). The late-stage tumor bearing samples were characterized by an increased distribution of the CNVs on the same chromosomes as well as several additional locations (Figure 4A). We then calculated a “total CNV score” across the genome by calculating the measured proportion of the chromosome containing CNV (a number between 0 to 1.0) and adding the values for all the chromosomes. The cluster distribution of the proportion of the genome containing CNV was then visualized using UMAPs, which revealed the highest “total CNV score” in the same clusters identified as “tumors” in early- (Figure 4B) and late-stage tumor-bearing samples (Figure 4C). A violin plot further showed that the highest enrichment for “total CNV score” was detected for the tumor clusters (Figures 4D,E).

Thus, the inferCNV analysis provided further support to the identification of “OPC-like” clusters as glioma cells in early- and late-stage tumor bearing brain tissue samples.

### Tumor cells from late-stage gliomas are enriched in genes involved in cell cycle, and gliogenesis

**Figure 5:**
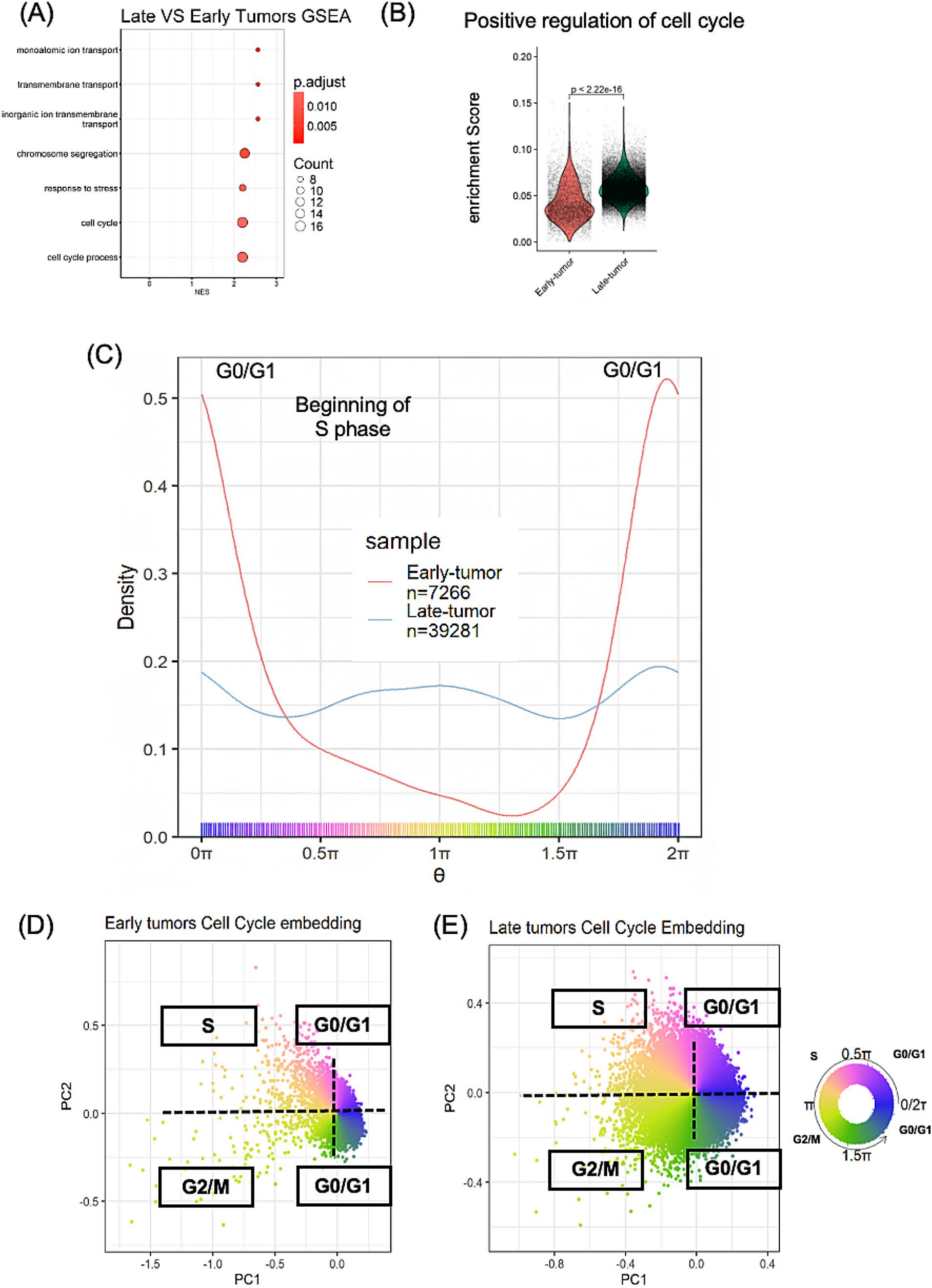
Late-stage tumor samples are characterized by a higher proportion of proliferating cells expressing genes involved in the positive regulation of the cell cycle. **(A)** Dotplot of the gene ontology categories differentially expressed between the filtered out “tumor clusters” from the three early-stage and three late-stage tumor-bearing samples. The X-axis shows the normalized enrichment scores (NES), and the dot size represents gene number. Please refer also to Supplementary Tables 4, 5. **(B)** Violin plot showing enrichment scores for curated gene set related to the positive regulation of the cell cycle. The indicated *p* value was calculated by Student’s *t*-test. **(C)** Visualization of the results of the Tricycle analysis. The Y-axis represents the fraction of cells relative to the total population (density). The X-axis represents the different phases of the cell cycle, with 0.5*π* to π representing cells in S phase and π to 1.5 π representing cells transitioning between G2/M. Cells within 0 to 0.5π or 1.5π to 2π are considered in G1 or G0. The red line represents the early-stage tumor cells density, with very few cells in S phase. The blue line represents the late-stage tumor cells, with a similar distribution in all the phases of the cell cycle. **(D,E)** PCA plots derived from Tricycle analysis. PCA coordinates indicate the cell cycle position and are annotated according to the color code shown on the right.

**Figure 6:**
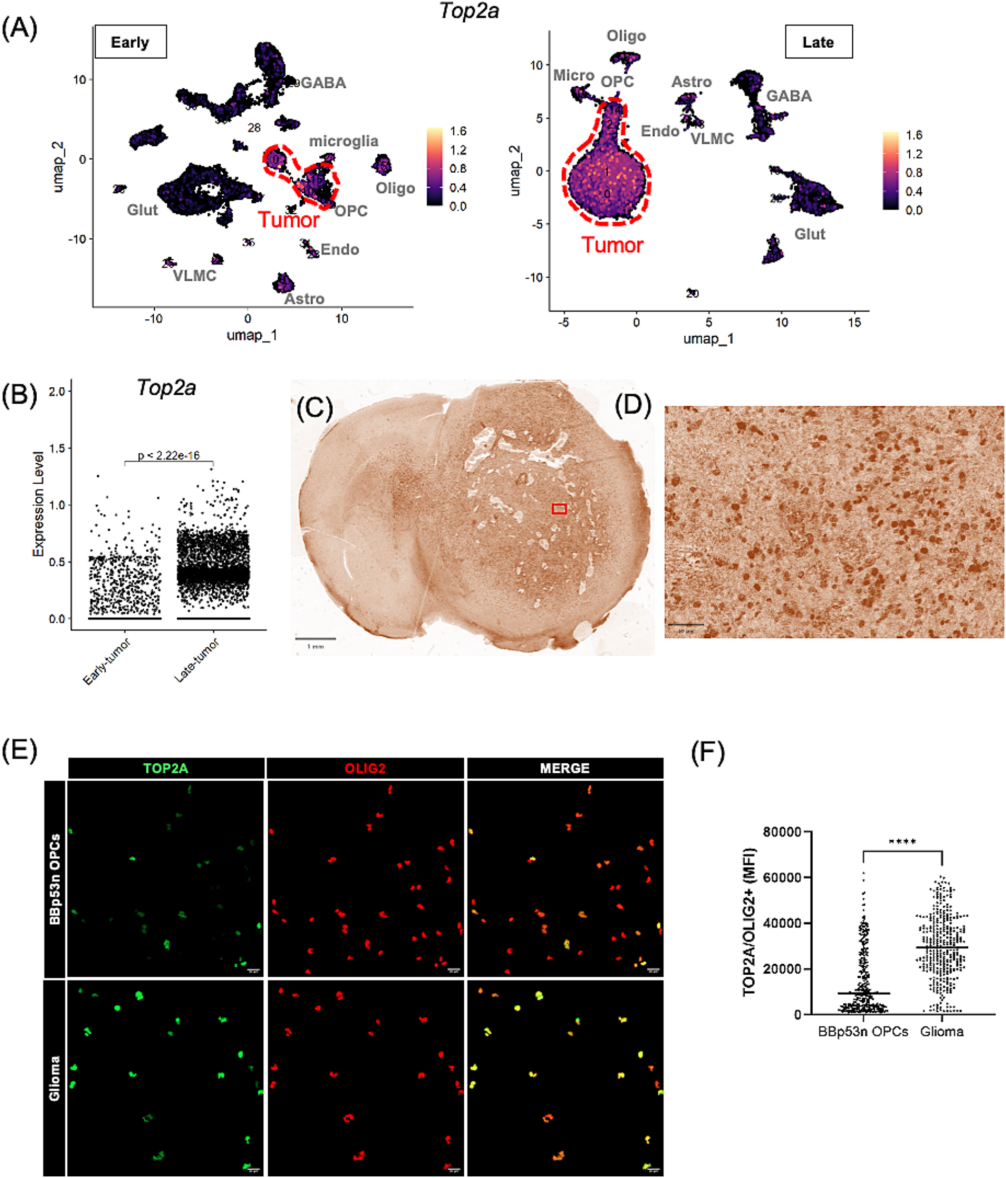
TOP2A expression is increased *in vivo* and in cultured glioma cells. **(A)** UMAP plots of early and late samples integrated with normal brain datasets, visualizing gene expression levels of *Top2a*. “Tumor’ cell clusters identified by the unique transcriptional signature are surrounded by a red dotted line. **(B)** Scatter plot illustrating the normalized expression levels of *Top2a* in early- and late-stage tumors from snRNA-seq data. *p*-value < 2.22e-16 calculated by Student’s *t*-test. **(C,D)** Representative images of immunohistochemical staining of TOP2a in brain sections of late-stage tumor samples at low **(C)** and high **(D)** magnification. Scale bars: 1 mm **(C)** and 50 μm **(D)**. **(E)** Representative confocal images of BB-p53n OPCs and glioma cells in culture, stained for TOP2a (green) and OLIG2 (red). Scale bar = 20 μm. **(F)** Scatterplot of the mean fluorescent intensity of TOP2a in OLIG2 + cells. *p*-value < 0.0001 calculated by Student’s *t*-test. Six to 12 images were quantified from each of three distinct experiments.

**Figure 7:**
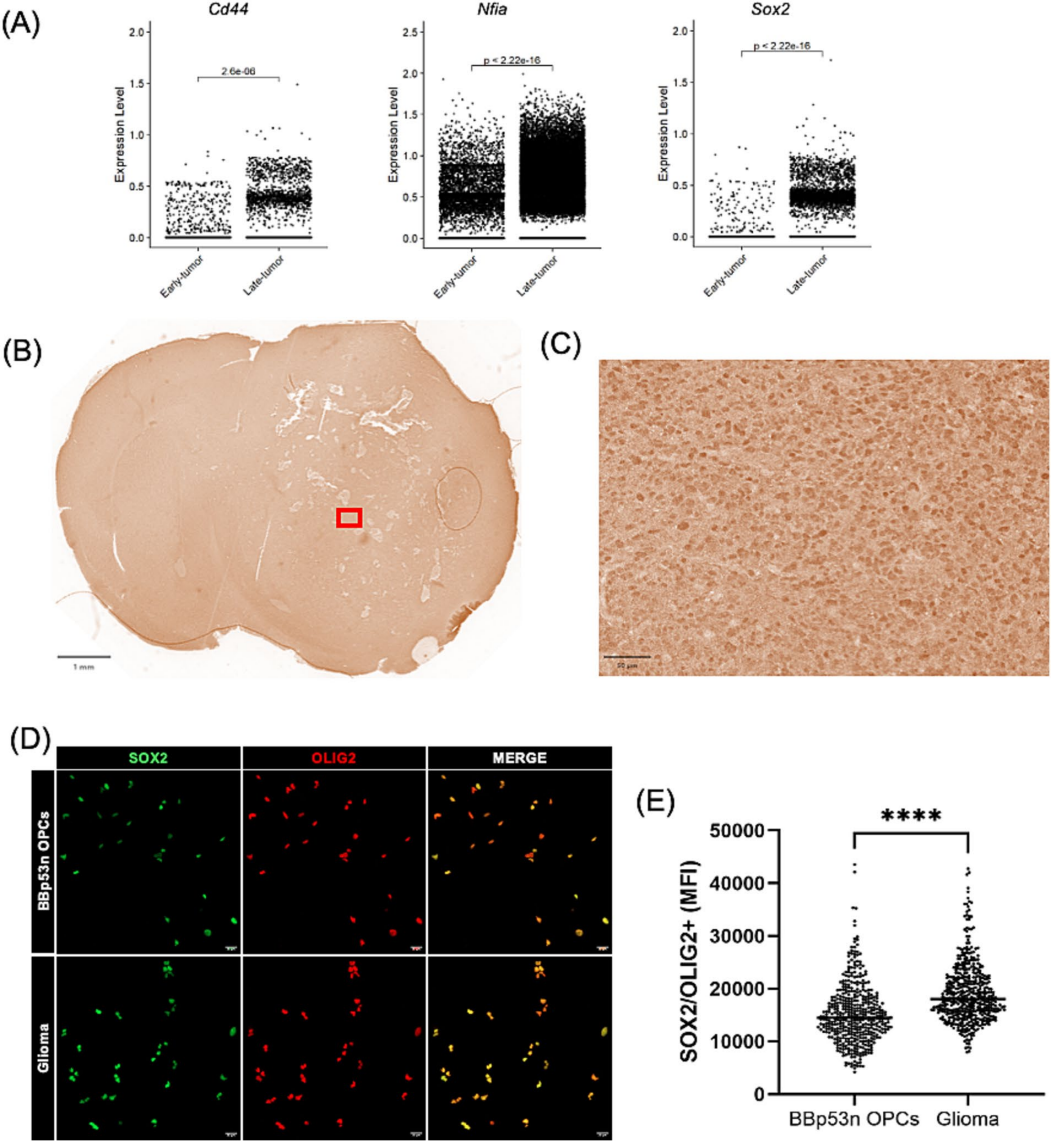
SOX2 expression is increased *in vivo* and in cultured glioma cells. **(A)** Scatter plots illustrating the normalized expression levels of transcript for *Cd44*, *Nfia*, *Sox2* in early- and late-stage tumors from snRNA-seq data. *p*-values calculated by Student’s *t*-test. **(B,C)** Representative image of SOX2 immunohistochemical staining in brain sections from late-stage tumors at low **(B)** and high magnification **(C)**. Scale bars: 1 mm **(B)** and 50 μm **(C)**. **(D)** Representative confocal images of SOX2 (green) and OLIG2 (red) staining in BB-p53n OPCs and glioma cells in culture. Scale bar = 20 μm. **(E)** Scatterplot of quantified mean fluorescent intensity of SOX2. *p*-value < 0.0001 calculated by Student’s *t*-test. Six to 12 images were quantified from each of three distinct experiments.

After filtering out the data from these “tumor” clusters from the three early-stage and three late-stage tumor-bearing brain tissue samples, we conducted a new differential expression (Supplementary Table 4) and GSEA (Supplementary Table 5) analysis to compare the late and early datasets, and interrogate the transcriptional changes occurring in the tumor cell clusters over time. The analysis revealed that late-stage glioma samples were enriched in gene ontology categories defined as positive regulation of the cell cycle (Figure 5A). More specifically, compared to early-stage, the late-stage tumor clusters were characterized by significantly greater levels of the transcriptional signatures (*p*-value < *2.2e-16*, Student’s t-test) for “positive regulation of the cell cycle” (Figure 5B). The enrichment of cell cycle related ontologies in the late-tumor samples led us to investigate the composition of cycle stages in both early- and late-stage tumors by implementing the TRICYCLE analysis (Zheng et al., 2022) which allows, through transcriptional profiling to identify the cell cycle phase where the cells were transiting at time of collection. TRICYCLE assigns a value between 0 and 2*π* to each nucleus, representing an estimation of the cell cycle position (G1/G0, S, G2, M). Nuclei with a value of 0–0.5π or 1.5π-2π identify nuclei closer to the G1/G0 transition, those with values between 0.5π and π identify nuclei in S phase and those between π and 1.5π nuclei in G2/M phase (Figure 5C). This analysis revealed that early-stage tumors were characterized by a high proportion of cells arrested at the G1/G0 transition and fewer than 10% of the cells in the S to G2/M phases (Figure 5D). Since the G1 phase transition is characterized by the presence of the restriction point when cells become committed to cell division, these data suggested that early-stage samples were characterized by cells with a proliferative advantage that still retained the ability to stop at the G1 checkpoint. Late-stage tumor cells, in contrast, lost this distribution and were characterized by closer to equal fractions of cells at each stage of the cell cycle, suggestive of loss of cell cycle control (Figure 5E). The detection of higher expression levels of *Mki67* and *Myc* in the late-stage tumor-bearing brain tissue samples, compared to the early-stage ones (Supplementary Figure 7A) further supported the TRICYCLE results. This observation was also supported by data in cultured cells revealing a greater proportion of cycling cells in glioma cells compared to mutant BB-p53n OPCs (Supplementary Figures 7B,C). Among the transcripts characterizing this progressive expansion of the proliferative population, *Top2a* was initially detected in the small tumor cluster in the early-stage tumor bearing samples and later found in almost every cell of the larger tumor cluster in late-stage tumor bearing samples (Figure 6A). Indeed, higher levels of *Top2*a transcripts were detected in the late brain samples compared to the early ones (Figure 6B) and immunohistochemical analysis confirmed the presence of a large number of TOP2A + cells in the tumor (Figures 6C,D). Cultured glioma cells were also characterized by higher TOP2A protein levels (Figures 6E,F) than mutant BB-p53n OPCs. Transcripts related to gliogenesis such as the cell surface protein *Cd44* (Senbanjo and Chellaiah, 2017) and the transcription factors *Sox2* and *Nfia,* were also higher in late-stage compared to early-stage tumor-bearing samples (Figure 7A). The increased levels of SOX2 protein were also detected in brain sections from late-stage tumors by immunohistochemistry (Figures 7B,C) and was consistent with the increased SOX2 + immunoreactivity detected in cultured glioma cells compared to mutant BB-p53n OPCs (Figures 7D,E). Together, these data suggest that the glioma cellpopulation is characterized by increased proliferation and features of primitive OPCs.

### Pseudo-time analysis on cycling tumor cells reveals a transcriptional evolution characterized by alterations in genes involved in cell cycle control and glutamate receptor signaling

**Figure 8:**
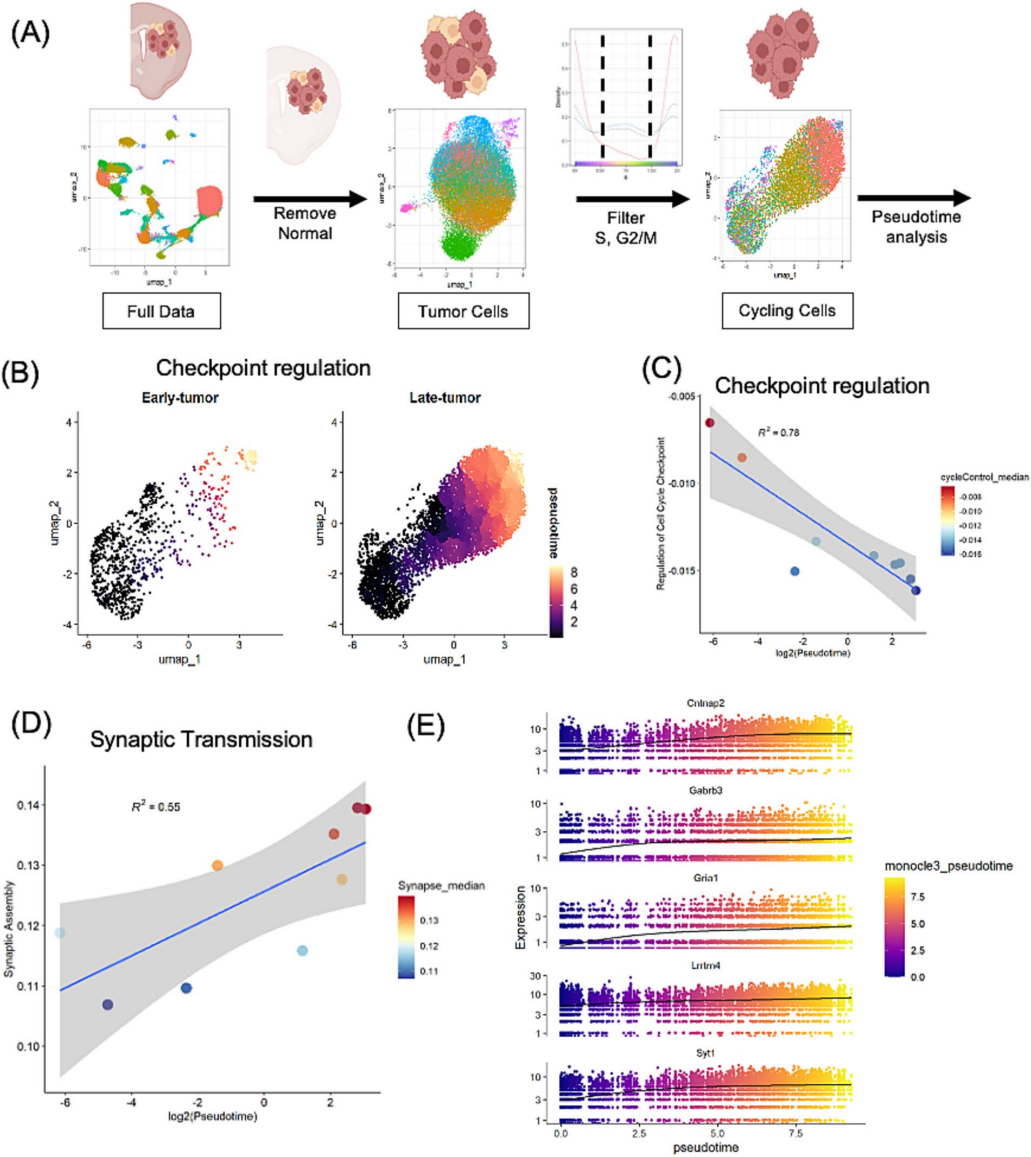
Actively dividing tumor cells show a developmental trajectory of decreasing cell cycle checkpoint regulation and increasing post-synaptic signaling. **(A)** Scheme of the process of filtering early- and late-stage data for pseudotime analysis. **(B)** UMAP plots of tumor cells in the S and G2/M phases split by early- and late-stage tumor origins. Point colors represent inferred pseudotime values by monocle3. **(C)** Cell cycle checkpoint regulation enrichment scores decreasing across pseudotime. Linear regression line in blue. Y-axis displays enrichment scores of GO database ‘Regulation of cell cycle checkpoint’ ontology. X-axis displays the log_2_ transformed pseudotime values. Point color represents median enrichment scores in legend. **(D)** Synaptic assembly enrichment scores increasing across pseudotime. Linear regression line in blue. Y-axis displays enrichment scores of GO database ‘Synaptic assembly’ ontology. X-axis displays the log_2_ transformed pseudotime values. Point color represents median enrichment scores in legend. **(E)** Scatterplots with trend lines representing the increased expression of the indicated transcripts over pseudotime.

To further characterize transcriptional changes occurring during the transition from early- to late-stage samples, we conducted a pseudo-time analysis, which represents a computational inference of transcriptional distance and cell-state transition (Trapnell et al., 2014). Since this study addresses tumor formation after injection of mutant OPCs, characterized by the presence of an “OPC-like” signature, it was biologically sensible to select this population as the root for the analysis. To define a “trajectory,” we selected the “tumor clusters” further filtered for nuclei estimated to be in S, G2, or M phases by TRICYCLE analysis, and this subset of cycling tumor cells was analyzed using the monocle3 algorithm and assign pseudo-time values (Figure 8A). We first noticed that there was a higher fraction of cycling cells in the late-stage samples as the pseudo-time showed an upward trend or, in other words, they dominated the later parts of the inferred pseudo-time trajectory (Figure 8B). Of note, we repeated the analysis including also non-cycling cells, and this led to unreasonable trajectories and produced inconclusive results. This was consistent with the fact that the Pearson’s correlation coefficient between pseudo-time and gene expression was the highest when only cycling cells were considered (Supplementary Figure 8). To investigate any pseudo-temporal changes in biological processes, we related enrichment scores to pseudo-time and identified decreased GO categories related to “cell cycle checkpoint regulation” (GO:0000075) across the trajectory (Figure 8C). In addition, we also observed an increase in synaptic assembly genes (GO:0007215) across the trajectory (Figure 8D). To directly investigate the positive or negative correlation between pseudo-time and gene expression, a Pearson correlation coefficient was calculated for each gene to examine if there were expression signatures increasing or decreasing along the computationally simulated pseudo-time values (Supplementary Table 6). Among the most positively correlated genes within the synaptic transmission gene ontology, we detected genes such as *Lrrtm4* (Pearson’s coefficient = *0.32*), encoding a protein critical for the assembly of glutamatergic synapses on central neurons (Zhou et al., 2018; Senbanjo and Chellaiah, 2017) and of GABAergic synapses in the retina (Siddiqui et al., 2013), those encoding for presynaptic proteins, such as *Syt1* (Pearson’s coefficient = *0.49*) and *Cntnap2* (Pearson’s coefficient = *0.56*), and for the post-synaptic glutamate ionotropic receptor AMPA type subunit 1 *Gria1* (Pearson’s coefficient *0.28*) and the GABAergic receptor subunits, such as the GABA Type A receptor subunit gamma3, encoded by *Gabrg3* (Pearson’s coefficient 0.28), beta 2 and beta 3, encoded by *Gabrb2* and *Gabrb3*, respectively (Figure 8E). Of relevance, these synaptic-transmission genes mapped mostly to neuronal populations in early-brain samples and were particularly enriched in the tumor cluster in the late-brain samples (Supplementary Figure 9), providing evidence of transcriptional support for synaptic engagement, consistent with the current literature.

**Figure 9:**
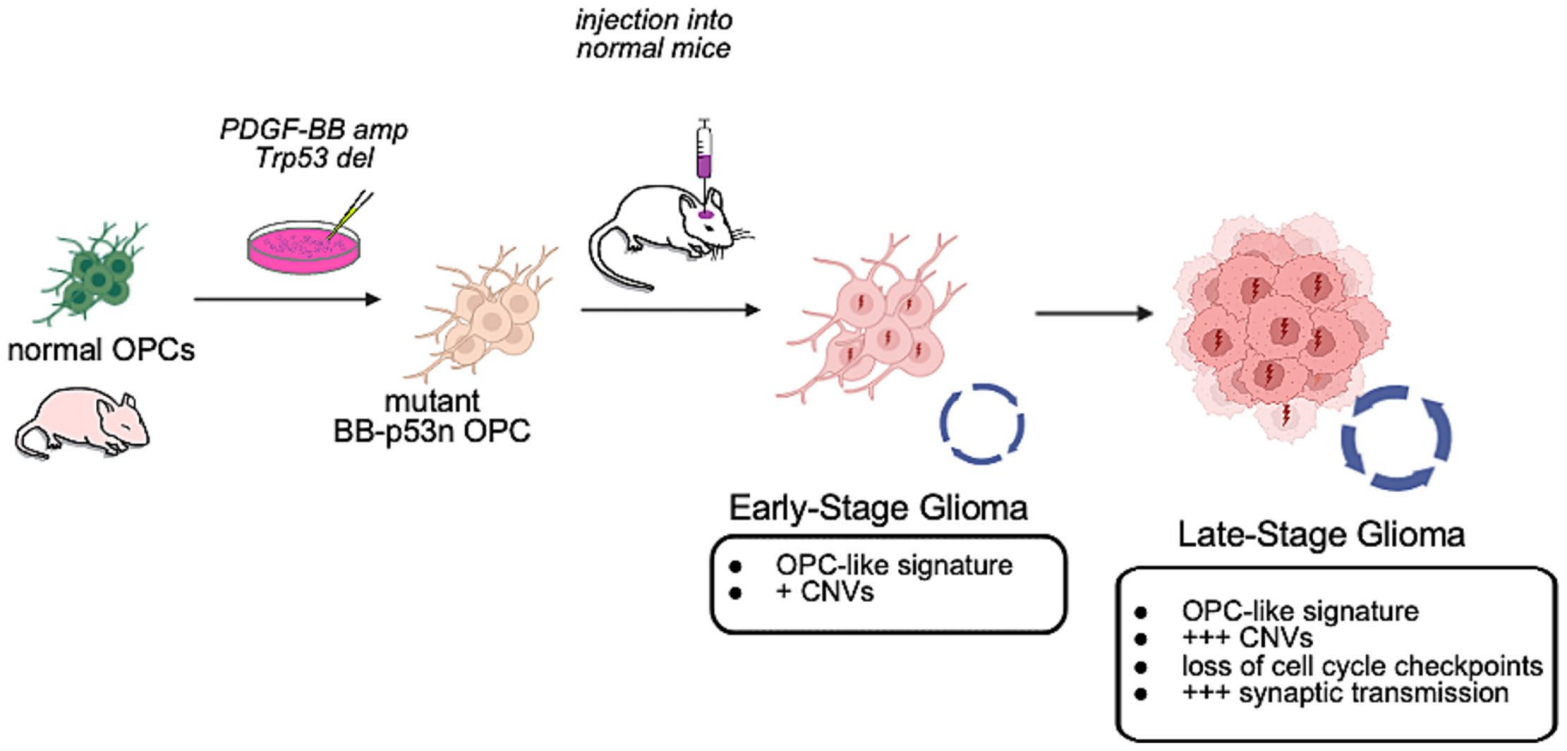
Schematics of the model proposed in this manuscript. The model shown here present the methodology used in this study and its main findings. Briefly, mutant OPCs, overexpressing PDGF-BB and carrying a *Trp53* deletion were generated *in vitro*, using a retroviral approach. Upon injection of these cells into normal recipient mice, we harvested brain tissue at early and late time points, corresponding to the detection of small and large tumoral masses by MRI and processed them for single nucleus RNA sequencing. The tumor clusters at early stages, were characterized by the presence of a unique OPC-like signature, the presence of CNVs (thunderbolds) and proliferation (blue circular arrows). Those at the late stages, were characterized by a similar transcriptional signature, more widespread distribution of the CNVs, loss of cell cycle check points and transcriptional evidence of increased synaptic transmission.

Overall, these data support a step-wise model of gliomagenesis after the injection of mutant OPCs, consistent with the detection of a cluster of highly proliferative cells, with genome-wide distribution of inferred CNVs at the early stages, followed by dramatic expansion, accrual of additional CNVs and a transcriptional signature consistent with the loss of cell cycle checkpoints and the enrichment of synaptic-transmission, likely due to enhanced neuro-glioma interaction (Figure 9).

## Discussion

We previously suggested that the gliomagenesis resulting from the *in vivo* injection of BB-p53n OPCs, and not by p53n OPCs, into the subcortical white matter of syngeneic recipient mice was likely due to transcriptional and epigenetic differences between these two cell types (Huang et al., 2024). However, it remained unclear whether the mutant BB-p53n OPCs represented a homogeneous or heterogeneous population and what transcriptional changes occurred following their injection into the brain as glioma formation progressed. These questions have been addressed in this manuscript, by combining the analysis of snRNA-seq on the BB-p53n mutant OPCs with that of brain samples, longitudinally collected at two time points after their injection into recipient mice and containing early- and late-stage tumors, as detected by MRI. The results on the BB-p53n OPCs identified a population characterized by OLIG2 and PDGFRα immunoreactivity and devoid of contaminating cells from other lineages. The BB-p53n OPCs, however, represented a transcriptionally heterogeneous population, inclusive of clusters enriched for the expression of gliogenic transcription factors (e.g., *Sox2* and *Nfia*) and others characterized by low levels of expression of the gene encoding for the neural progenitor βIII tubulin cytoskeletal protein *Tubb3,* although the complete Neftel’s NPCs signature (Neftel et al., 2019) was not enriched in any of the identified clusters. The presence of clusters enriched for *Nfia*, previously reported to support glioma survival and growth (Lee et al., 2017; Lee et al., 2014) and for the stem cell marker *Sox2* (Driessens and Blanpain, 2011; Ellis et al., 2004), previously reported to contribute to the invasive growth of gliomas (Alonso et al., 2011), was reminiscent of primitive OPCs, which were previously suggested as the cell-of-origin of glioma (Weng et al., 2019).

After the injection of mutant BB-p53n OPCs into the subcortical white matter of recipient mice, we longitudinally collected brain samples at the first signs of detection of tumoral masses and at later time points. The novelty of this manuscript is in characterizing the longitudinal progression from injected BB-p53n OPCs to late-stage tumors. When the snRNA-seq data from the longitudinally collected samples were integrated with a normal brain dataset, we identified several cell clusters corresponding to different cell types, as well as one cluster that was not found in the normal brain dataset. This cluster was enriched for OPC and glioma markers and was annotated as “OPC-like.” Of relevance, the “OPC-like” cluster was also characterized by the expression of positive regulators of the cell cycle and transcripts related to gliogenesis, reminiscent of the signature of primitive OPCs, and likely reflecting the expansion of the injected BB-p53n OPCs. The analysis of the early-stage tumor-bearing samples led to the identification of several normal brain cell clusters consistent with the interpretation that the small tumoral lesions had not severely affected the local parenchyma, as revealed also by MRI imaging and by the detection of high gene counts per nucleus in those samples. The analysis of the late-stage tumor bearing samples, in contrast, was characterized by the presence of large tumor clusters associated with lower gene counts per nucleus, the depletion of normal brain cell clusters, consistent with the detection of massive tumors by MRI and the extensive destruction of the parenchyma and presence of large necrotic areas by histopathology.

When we integrated the normal brain dataset either with the three early-stage glioma -containing brain samples or with the three late-stage glioma -containing samples, we identified clusters that were not found in the normal brain and that were enriched for the OPC-like signature as well as for the Neftel’s OPC and NPC signature. We identified these unique clusters as “tumors” as they were also enriched for glioma markers, such as *Top2a* and *My*c. These clusters were smaller in the samples collected at the early time-point after injection and much larger in the samples collected at late time points. Inferred CNV analysis of these early- and late-stage samples further revealed a progressive spreading of the CNVs across the genome. While CNVs can be detected in the normal brain, they are generally confined to few genes and confined to regions less than one Mb in size (Rohrback et al., 2018). In contrast, the CNVs in tumors are typically detected in much wider genomic regions (Khalil et al., 2020). Consistent with the biological relevance for glioma, we detected amplifications in chromosome 1, the most gene-dense region of the mouse genome and also a genetic hallmark of gliomas (Buchwald et al., 2020) and chromosomal instability (Zhao et al., 2019). The samples collected at the late-stages after injection were characterized by a more widespread chromosomal distribution of inferred CNVs than the early-stage samples. Quantification of the total CNV load score, followed by UMAP distribution, further allowed us to identify the clusters with the highest CNV score as the ones enriched for the OPC-like and primitive OPC signature. Since our transcriptomic analysis was conducted on nuclei collected at early and late time points after injection of mutant OPCs carrying both an oncogenic stimulus (PDGF-BB) and the loss of *Trp53*, obtained through retroviral transformation of isolated OPCs, we interpreted these data as suggestive of the result of subsequent rapid expansion driven by the proliferation of these pre-transformed cells.

When we attempted to define an ordered trajectory by centering the root to the OPC-like signature cluster and performed a pseudotime analysis of the data from cycling cells from early- and late-stage tumor-bearing brain samples, we further identified decreasing levels of transcripts related to cell cycle checkpoints and increasing levels of synaptic transmission genes. Checkpoint deregulation has long been implicated in both therapeutic strategies and acquired resistances as it is feature of aggressive glioma growth (Vanan et al., 2012; Tachon et al., 2018). The “synaptic transmission” gene ontology category reflected increased genes encoding for proteins involved in pre- and post-synaptic compartments. Among the pre-synaptic genes we detected *Syt1*, encoding for synaptotagmin 1 and the gene *Cntnap2*, encoding the presynaptic adhesion molecule CASPR 2, important for neuro-glia interactions. Among the post-synaptic genes we detected: *Gria1*, encoding for the AMPA receptor subunit; *Gabrb3,* encoding for the GABA receptor subunits beta 3 and *Lrrtm4* which encodes a component of postsynaptic ligands for the neurexins, previously reported as mediators of neuro/glioma interactions (Venkatesh et al., 2015) and reported to regulate the assembly of glutamatergic (Siddiqui et al., 2013; De Wit et al., 2009) and GABAergic synapses (Sinha et al., 2020). *Gria1 and Gabrb3* are of particular relevance in light of the previously reported importance of AMPA-driven signaling (Venkataramani et al., 2019) and the recent report of GABAergic synapses in high grade glioma (Barron et al., 2025).

These results are consistent with previous transcriptomics and ultrastructural studies of adult and pediatric gliomas demonstrating that neurons establish *bona-fide* synaptic contacts onto glioma cells and enhance proliferation by inducing their depolarization (Venkatesh et al., 2019). They are also consistent with previous literature demonstrating glutamatergic (Venkataramani et al., 2019; Venkataramani et al., 2022) and GABAergic (Barron et al., 2025) signaling on glioma cells. However, while our data provide further transcriptional support to the importance of synaptic transmission during tumor progression, further validation via immunostaining for pre- and post-synaptic markers and functional assays is necessary to definitively establish the significance of these findings.

In conclusion, this study on tumor formation and progression after the injection of mutant OPCs in the brain parenchyma (Figure 9), using a longitudinal single-nucleus RNA-seq analysis highlights the importance of the expansion of cell clusters with an “OPC-like” signature characterized by primitive OPC gene expression, likely derived from the injected mutant BB-p53n OPCs during the early stages of tumor formation. Due to the high level of genes regulating cell division in these cells, characterized by an “OPC-like” signature, reminiscent of primitive OPCs, we propose that these cells are the ones that tend to expand and lead to the formation of tumor clusters. These clusters are characterized by the presence of CNVs, likely linked to the presence of amplifications and duplications in genes related to cell divisions (e.g., *Myc* and related pathways). It is likely that the tumors continue to expand in late-stage gliomas, in part due to the increasing genomic instability and checkpoint failure, in part due to the potentiation of the post-synaptic response associated with neuronal infiltration, leading to large masses which significantly disrupt the entire brain parenchyma. Overall, our data are consistent with previous literature on glioma formation and progression and identify potential vulnerabilities that could be exploited for therapeutic intervention.

## Data Availability

The datasets presented in this study can be found in online repositories. The names of the repository/repositories and accession number(s) can be found in the article/Supplementary material. Data deposited in GEO n.GSE309333. Code: https://github.com/dennishuang02/BBp53n_glioma_submission/tree/main.

## References

[ref1] Alonso M. M. Diez-Valle R. Manterola L. Rubio A. Liu D. Cortes-Santiago N. . (2011). Genetic and epigenetic modifications of Sox2 contribute to the invasive phenotype of malignant gliomas. PLoS One 6:e26740. doi: 10.1371/journal.pone.0026740, 22069467 PMC3206066

[ref2] Ashburner M. Ball C. A. Blake J. A. Botstein D. Butler H. Cherry J. M. . (2000). Gene ontology: tool for the unification of biology. Nat. Genet. 25, 25–29. doi: 10.1038/75556, 10802651 PMC3037419

[ref3] Barron T. Yalçın B. Su M. Byun Y. G. Gavish A. Shamardani K. . (2025). GABAergic neuron-to-glioma synapses in diffuse midline gliomas. Nature 639, 1060–1068. doi: 10.1038/s41586-024-08579-3, 39972132 PMC11946904

[ref4] Bergles D. E. Richardson W. D. (2016). Oligodendrocyte development and plasticity. Cold Spring Harb. Perspect. Biol. 8:a020453. doi: 10.1101/cshperspect.a020453, 26492571 PMC4743079

[ref5] Buchwald Z. S. Tian S. Rossi M. Smith G. H. Switchenko J. Hauenstein J. E. . (2020). Genomic copy number variation correlates with survival outcomes in WHO grade IV glioma. Sci. Rep. 10:7355. doi: 10.1038/s41598-020-63789-9, 32355162 PMC7192941

[ref6] Butler A. Hoffman P. Smibert P. Papalexi E. Satija R. (2018). Integrating single-cell transcriptomic data across different conditions, technologies, and species. Nat. Biotechnol. 36, 411–420. doi: 10.1038/nbt.4096, 29608179 PMC6700744

[ref7] Cencioni C. Scagnoli F. Spallotta F. Nasi S. Illi B. (2023). The ‘Superoncogene’ Myc at the crossroad between metabolism and gene expression in glioblastoma multiforme. Int. J. Mol. Sci. 24:4217. doi: 10.3390/ijms24044217, 36835628 PMC9966483

[ref8] De Wit J. Sylwestrak E. O’Sullivan M. L. Otto S. Tiglio K. Savas J. N. . (2009). LRRTM2 interacts with Neurexin1 and regulates excitatory synapse formation. Neuron 64, 799–806. doi: 10.1016/j.neuron.2009.12.019, 20064388 PMC2829666

[ref9] Driessens G. Blanpain C. (2011). Long live Sox2: Sox2 lasts a lifetime. Cell Stem Cell 9, 283–284. doi: 10.1016/j.stem.2011.09.007, 21982223

[ref10] Ellis P. Fagan B. M. Magness S. T. Hutton S. Taranova O. Hayashi S. . (2004). SOX2, a persistent marker for multipotential neural stem cells derived from embryonic stem cells, the embryo or the adult. Dev. Neurosci. 26, 148–165. doi: 10.1159/000082134, 15711057

[ref11] Erickson A. He M. Berglund E. Marklund M. Mirzazadeh R. Schultz N. . (2022). Spatially resolved clonal copy number alterations in benign and malignant tissue. Nature 608, 360–367. doi: 10.1038/s41586-022-05023-2, 35948708 PMC9365699

[ref12] Hao Y. Hao S. Andersen-Nissen E. Mauck W. M. Zheng S. Butler A. . (2021). Integrated analysis of multimodal single-cell data. Cell 184, 3573–3587.e29. doi: 10.1016/j.cell.2021.04.048, 34062119 PMC8238499

[ref13] Huang D. Mela A. Bhanu N. V. Garcia B. A. Canoll P. Casaccia P. (2024). PDGF-BB overexpression in p53 null oligodendrocyte progenitors increases H3K27me3 and induces transcriptional changes which favor proliferation. Neoplasia 57:101042. doi: 10.1016/j.neo.2024.101042, 39216363 PMC11402553

[ref14] inferCNV. (2025). *inferCNV of the Trinity CTAT Project*. *inferCNV of the Trinity CTAT Project*. Available online at: https://github.com/broadinstitute/inferCNV.

[ref15] Khalil A. I. S. Khyriem C. Chattopadhyay A. Sanyal A. Hierarchical discovery of large-scale and focal copy number alterations in low-coverage Cancer genomes. BMC Bioinformatics 21, 2020:147. doi: 10.1186/s12859-020-3480-3, 32299346 PMC7160937

[ref16] Komori T. (2022). The 2021 WHO classification of tumors, 5th edition, central nervous system tumors: the 10 basic principles. Brain Tumor Pathol. 39, 47–50. doi: 10.1007/s10014-022-00428-3, 35316415

[ref17] Lee J. S. Hoxha E. Song H. R. (2017). A novel NFIA-NFκB feed-forward loop contributes to glioblastoma cell survival. Neuro-Oncology 19, 524–534. doi: 10.1093/neuonc/now233, 27994064 PMC5473440

[ref18] Lee J. S. Xiao J. Patel P. Schade J. Wang J. Deneen B. . (2014). A novel tumor-promoting role for nuclear factor IA in glioblastomas is mediated through negative regulation of P53, P21, and PAI1. Neuro-Oncology 16, 191–203. doi: 10.1093/neuonc/not167, 24305710 PMC3895381

[ref19] Lei L. Sonabend A. M. Guarnieri P. Soderquist C. Ludwig T. Rosenfeld S. . (2011). Glioblastoma models reveal the connection between adult glial progenitors and the proneural phenotype. PLoS One 6:e20041. doi: 10.1371/journal.pone.0020041, 21625383 PMC3100315

[ref20] Liu C. Sage J. C. Miller M. R. Verhaak R. G. W. Hippenmeyer S. Vogel H. . (2011). Mosaic analysis with double markers (MADM) reveals tumor cell-of-origin in glioma. Cell 146, 209–221. doi: 10.1016/j.cell.2011.06.014, 21737130 PMC3143261

[ref21] Louis D. N. Perry A. Wesseling P. Brat D. J. Cree I. A. Figarella-Branger D. . (2021). The 2021 WHO classification of Tumors of the central nervous system: a summary. Neuro-Oncology 23, 1231–1251. doi: 10.1093/neuonc/noab106, 34185076 PMC8328013

[ref22] McKenzie A. T. Wang M. Hauberg M. E. Fullard J. F. Kozlenkov A. Keenan A. . (2018). Brain cell type specific gene expression and co-expression network architectures. Sci. Rep. 8:8868. doi: 10.1038/s41598-018-27293-5, 29892006 PMC5995803

[ref23] Neftel C. Laffy J. Filbin M. G. Hara T. Shore M. E. Rahme G. J. . (2019). An integrative model of cellular states, plasticity, and genetics for glioblastoma. Cell 178, 835–849.e21. doi: 10.1016/j.cell.2019.06.024, 31327527 PMC6703186

[ref24] Ostrom Q. T. Gittleman H. Fulop J. Liu M. Blanda R. Kromer C. . (2015). CBTRUS statistical report: primary brain and central nervous system Tumors diagnosed in the United States in 2008-2012. Neuro Oncol. 17, iv1–iv62. doi: 10.1093/neuonc/nov189, 26511214 PMC4623240

[ref25] Rajendran S. Hu Y. Canella A. Peterson C. Gross A. Cam M. . (2023). Single-cell RNA sequencing reveals immunosuppressive myeloid cell diversity during malignant progression in a murine model of glioma. Cell Rep. 42:112197. doi: 10.1016/j.celrep.2023.112197, 36871221

[ref26] Rajesh Y. Pal I. Banik P. Chakraborty S. Borkar S. A. Dey G. . (2017). Insights into molecular therapy of glioma: current challenges and next generation blueprint. Acta Pharmacol. Sin. 38, 591–613. doi: 10.1038/aps.2016.167, 28317871 PMC5457688

[ref27] Robinson M. D. McCarthy D. J. Smyth G. K. (2010). edgeR: a Bioconductor package for differential expression analysis of digital gene expression data. Bioinformatics 26, 139–140. doi: 10.1093/bioinformatics/btp616, 19910308 PMC2796818

[ref28] Rohrback S. April C. Kaper F. Rivera R. R. Liu C. S. Siddoway B. . (2018). Submegabase copy number variations Arise during cerebral cortical neurogenesis as revealed by single-cell whole-genome sequencing. Proc. Natl. Acad. Sci. 115, 10804–10809. doi: 10.1073/pnas.1812702115, 30262650 PMC6196524

[ref29] Satija R. Farrell J. A. Gennert D. Schier A. F. Regev A. (2015). Spatial reconstruction of single-cell gene expression data. Nat. Biotechnol. 33, 495–502. doi: 10.1038/nbt.3192, 25867923 PMC4430369

[ref30] Schindelin J. Arganda-Carreras I. Frise E. Kaynig V. Longair M. Pietzsch T. . (2012). Fiji: an open-source platform for biological-image analysis. Nat. Methods 9, 676–682. doi: 10.1038/nmeth.2019, 22743772 PMC3855844

[ref31] Senbanjo L. T. Chellaiah M. A. (2017). CD44: a multifunctional cell surface adhesion receptor is a regulator of progression and metastasis of Cancer cells. Front. Cell Dev. Biol. 5:18. doi: 10.3389/fcell.2017.00018/full 28326306 PMC5339222

[ref32] Siddiqui T. J. Tari P. K. Connor S. A. Zhang P. Dobie F. A. She K. . (2013). An LRRTM4-HSPG complex mediates excitatory synapse development on dentate gyrus granule cells. Neuron 79, 680–695. doi: 10.1016/j.neuron.2013.06.029, 23911104

[ref33] Sinha R. Siddiqui T. J. Padmanabhan N. Wallin J. Zhang C. Karimi B. . (2020). LRRTM4: a novel regulator of presynaptic inhibition and ribbon synapse arrangements of retinal bipolar cells. Neuron 105, 1007–1017.e5. doi: 10.1016/j.neuron.2019.12.028, 31974009 PMC7165459

[ref34] Sonabend A. M. Yun J. Lei L. Leung R. Soderquist C. Crisman C. . (2013). Murine cell line model of proneural glioma for evaluation of anti-tumor therapies. J. Neuro Oncol. 112, 375–382. doi: 10.1007/s11060-013-1082-x, 23504257 PMC3694577

[ref35] Stuart T. Butler A. Hoffman P. Hafemeister C. Papalexi E. Mauck W. M. . (2019). Comprehensive integration of single-cell data. Cell 177, 1888–1902.e21. doi: 10.1016/j.cell.2019.05.031, 31178118 PMC6687398

[ref36] Subramanian A. Tamayo P. Mootha V. K. Mukherjee S. Ebert B. L. Gillette M. A. . (2005). Gene set enrichment analysis: a knowledge-based approach for interpreting genome-wide expression profiles. Proc. Natl. Acad. Sci. 102, 15545–15550. doi: 10.1073/pnas.0506580102, 16199517 PMC1239896

[ref37] Tachon G. Cortes U. Guichet P. O. Rivet P. Balbous A. Masliantsev K. . (2018). Cell cycle changes after glioblastoma stem cell irradiation: the major role of RAD51. Int. J. Mol. Sci. 19:3018. doi: 10.3390/ijms19103018, 30282933 PMC6213228

[ref38] Tirosh I. Venteicher A. S. Hebert C. Escalante L. E. Patel A. P. Yizhak K. . (2016). Single-cell RNA-seq supports a developmental hierarchy in human oligodendroglioma. Nature 539, 309–313. doi: 10.1038/nature20123, 27806376 PMC5465819

[ref39] Trapnell C. Cacchiarelli D. Grimsby J. Pokharel P. Li S. Morse M. . (2014). The dynamics and regulators of cell fate decisions are revealed by pseudotemporal ordering of single cells. Nat. Biotechnol. 32, 381–386. doi: 10.1038/nbt.2859, 24658644 PMC4122333

[ref40] Upadhyayula P. S. Higgins D. M. Mela A. Banu M. Dovas A. Zandkarimi F. . (2023). Dietary restriction of cysteine and methionine sensitizes gliomas to ferroptosis and induces alterations in energetic metabolism. Nat. Commun. 14:1187. doi: 10.1038/s41467-023-36630-w, 36864031 PMC9981683

[ref41] Vanan I. Dong Z. Tosti E. Warshaw G. Symons M. Ruggieri R. (2012). Role of a DNA damage checkpoint pathway in ionizing radiation-induced glioblastoma cell migration and invasion. Cell Mol. Neurobiol. 32, 1199–1208. doi: 10.1007/s10571-012-9846-y, 22552889 PMC11498580

[ref42] Venkataramani V. Tanev D. I. Strahle C. Studier-Fischer A. Fankhauser L. Kessler T. . (2019). Glutamatergic synaptic input to glioma cells drives brain tumour progression. Nature 573, 532–538. doi: 10.1038/s41586-019-1564-x, 31534219

[ref43] Venkataramani V. Yang Y. Schubert M. C. Reyhan E. Tetzlaff S. K. Wißmann N. . (2022). Glioblastoma hijacks neuronal mechanisms for brain invasion. Cell 185, 2899–2917.e31. doi: 10.1016/j.cell.2022.06.054, 35914528

[ref44] Venkatesh H. S. Johung T. B. Caretti V. Noll A. Tang Y. Nagaraja S. . (2015). Neuronal activity promotes glioma growth through Neuroligin-3 secretion. Cell 161, 803–816. doi: 10.1016/j.cell.2015.04.012, 25913192 PMC4447122

[ref45] Venkatesh H. S. Morishita W. Geraghty A. C. Silverbush D. Gillespie S. M. Arzt M. . (2019). Electrical and synaptic integration of glioma into neural circuits. Nature 573, 539–545. doi: 10.1038/s41586-019-1563-y, 31534222 PMC7038898

[ref46] Verhaak R. G. W. Hoadley K. A. Purdom E. Wang V. Qi Y. Wilkerson M. D. . (2010). Integrated genomic analysis identifies clinically relevant subtypes of glioblastoma characterized by abnormalities in PDGFRA, IDH1, EGFR, and NF1. Cancer Cell 17, 98–110. doi: 10.1016/j.ccr.2009.12.020, 20129251 PMC2818769

[ref47] Weng Q. Wang J. Wang J. He D. Cheng Z. Zhang F. . (2019). Single-cell transcriptomics uncovers glial progenitor diversity and cell fate determinants during development and Gliomagenesis. Cell Stem Cell 24, 707–723.e8. doi: 10.1016/j.stem.2019.03.006, 30982771 PMC6669001

[ref48] Wickham H. (2016). *ggplot2: Elegant Graphics for Data Analysis*. Available online at: https://ggplot2.tidyverse.org.

[ref49] Yao Z. Liu H. Xie F. Fischer S. Adkins R. S. Aldridge A. I. . (2021). A transcriptomic and epigenomic cell atlas of the mouse primary motor cortex. Nature 598, 103–110. doi: 10.1038/s41586-021-03500-8, 34616066 PMC8494649

[ref50] Yu G. (2024). *Enrichplot: Visualization of Functional Enrichment Result*. Available online at: https://yulab-smu.top/biomedical-knowledge-mining-book/.

[ref51] Yu G. Wang L. G. Han Y. He Q. Y. (2012). clusterProfiler: an R package for comparing biological themes among gene clusters. OMICS 16, 284–287. doi: 10.1089/omi.2011.0118, 22455463 PMC3339379

[ref52] Zhao Y. Carter R. Natarajan S. Varn F. S. Compton D. A. Gawad C. . (2019). Single-cell RNA sequencing reveals the impact of chromosomal instability on glioblastoma Cancer stem cells. BMC Med. Genet. 12:79. doi: 10.1186/s12920-019-0532-5, 31151460 PMC6545015

[ref53] Zheng S. C. Stein-O’Brien G. Augustin J. J. Slosberg J. Carosso G. A. Winer B. . (2022). Universal prediction of cell-cycle position using transfer learning. Genome Biol. 23:41. doi: 10.1186/s13059-021-02581-y, 35101061 PMC8802487

[ref54] Zhou T. Wang Y. Qian D. Liang Q. Wang B. (2018). Over-expression of TOP2A as a prognostic biomarker in patients with glioma. International journal of. Clin. Exp. Pathol. 11, 1228–1237, 31938217 PMC6958105

[ref55] Zong H. Verhaak R. G. Canoll P. (2012). The cellular origin for malignant glioma and prospects for clinical advancements. Expert Rev. Mol. Diagn. 12, 383–394. doi: 10.1586/erm.12.30, 22616703 PMC3368274

