# Supplementary material for: Single-nucleus transcriptomics identifies cell cycle and synaptic pathway dysregulation during OPC-to-glioma progression
Source: Front Cell Neurosci. 2026 Jun 30;20:1713437. doi: 10.3389/fncel.2026.1713437 (PMC13424661; doi:10.3389/fncel.2026.1713437)
Supplement: Supplementary file 1 [file Data_Sheet_1.pdf]

Supplemental material.

Single-nucleus brain transcriptomics after injection of mutant OPCs identifies dysregulation of cell cycle and synaptic pathways during the progression to gliomas.

Dennis Huang[1,2], Angeliki Mela[3], Hye-Jin Park[2], Peter Canoll[3], Patrizia Casaccia[1,2]

Data Availability
snRNA-seq datasets from BB-p53n OPCs and from brain tissue at early and late times after injections have been deposited in the NCBI's GEO depository and are available in raw and processed forms using the accession number GSE309333.
Example R code for analysis is available in the public github repository:
https://github.com/dennishuang02/BBp53n_glioma_submission/tree/main

Suppl. Figure 1

Suppl. Figure 2

Suppl. Figure 3

Suppl. Figure 4

Suppl. Figure 5

Suppl. Figure 6

Suppl. Figure 7

Suppl. Figure 8

Suppl. Figure 9

Table legends and related links

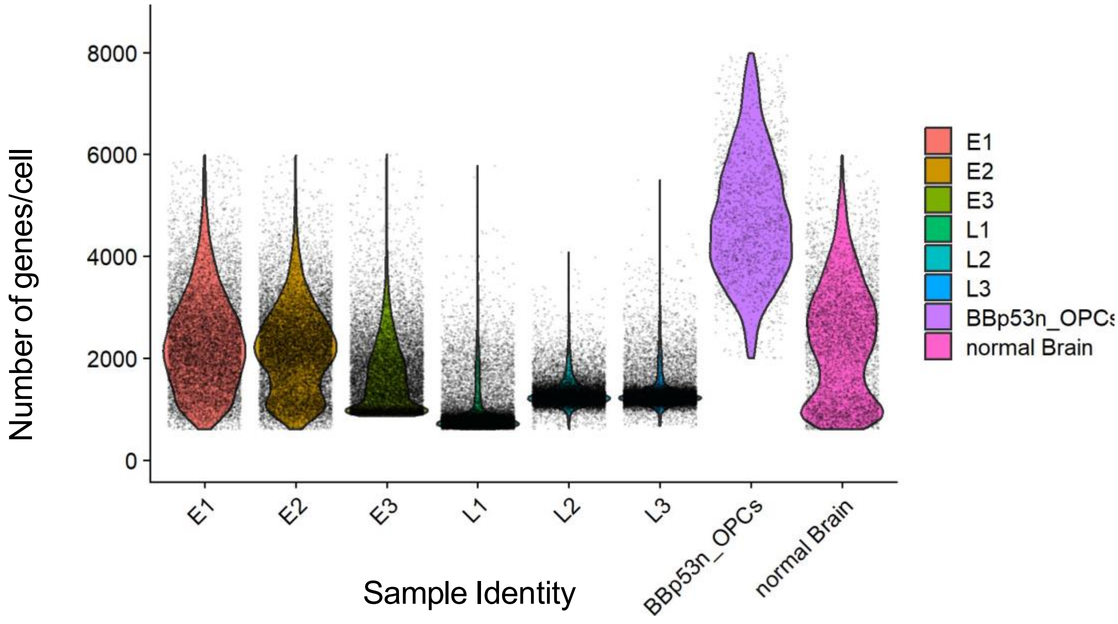

Supplemental Figure 1. QC using nFeature RNA function for all the samples analyzed.

Violin plot depicts the number of genes detected in each nucleus on the Y axis and the samples identity on the X axis. The distribution reflects higher feature counts in the datasets obtained from the cultured mutant OPCS (BBp53n_OPCs), the distribution for the normal brain dataset and the higher feature counts in the early-stage tumor bearing samples (E1,E2,E3) compared to the late-stage samples (L1, L2, L3), likely reflecting the extensive tissue damage and necrosis detected in the late-stage tumors.

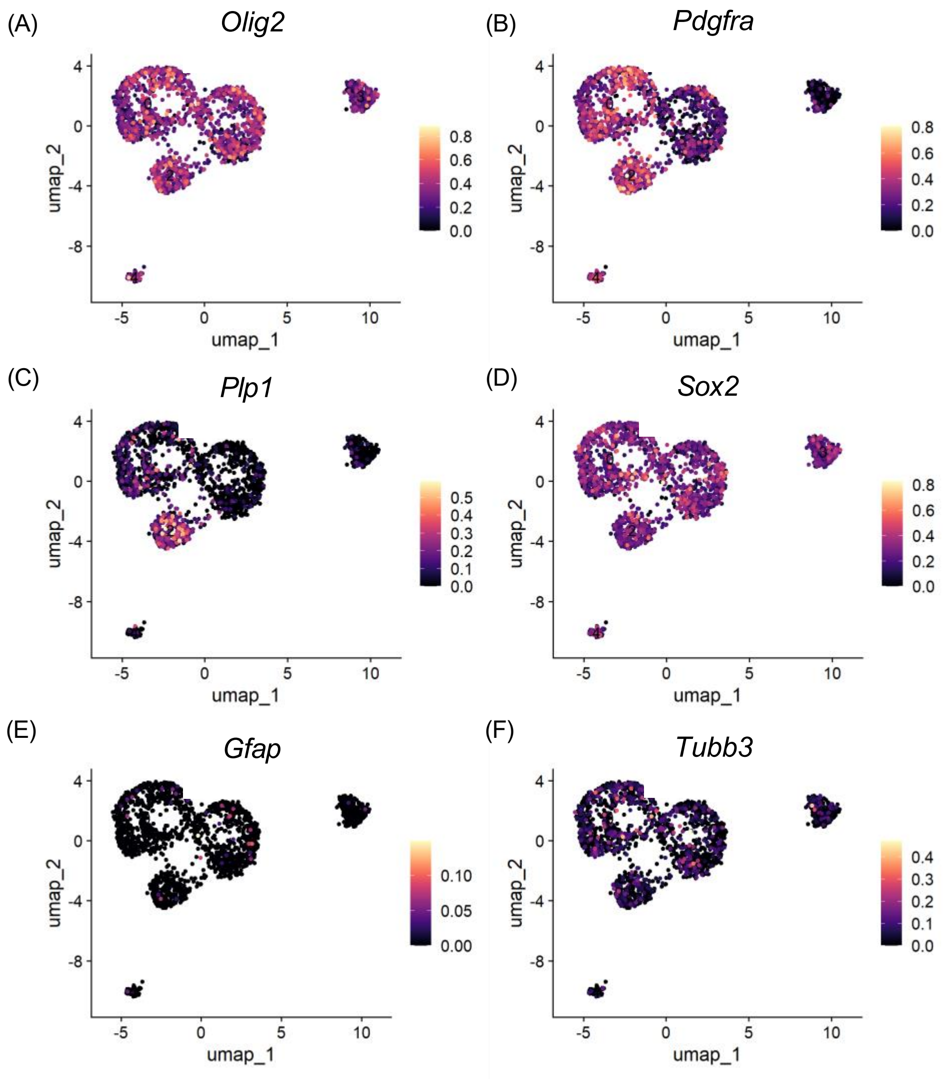

Supplemental Figure 2: BB-p53n OPCs show near homogenous expression of OPC markers and *Sox2* but few nuclei express markers of other cell-types

(A)-(F) UMAP plots of BB-p53n OPCs. The color gradient of the dots represents the relative expression levels of transcripts, with black dots denoting very low expression and red to yellow progressively higher expression. The cluster enrichment is shown for the OPC markers (*Olig2, Pdgfra)*(A-B), the oligodendrocyte marker *Plp1* (C), the stem cell marker *Sox2* (D), the astrocyte marker *Gfap* (E), and the neuronal marker *Tubb3* (F).

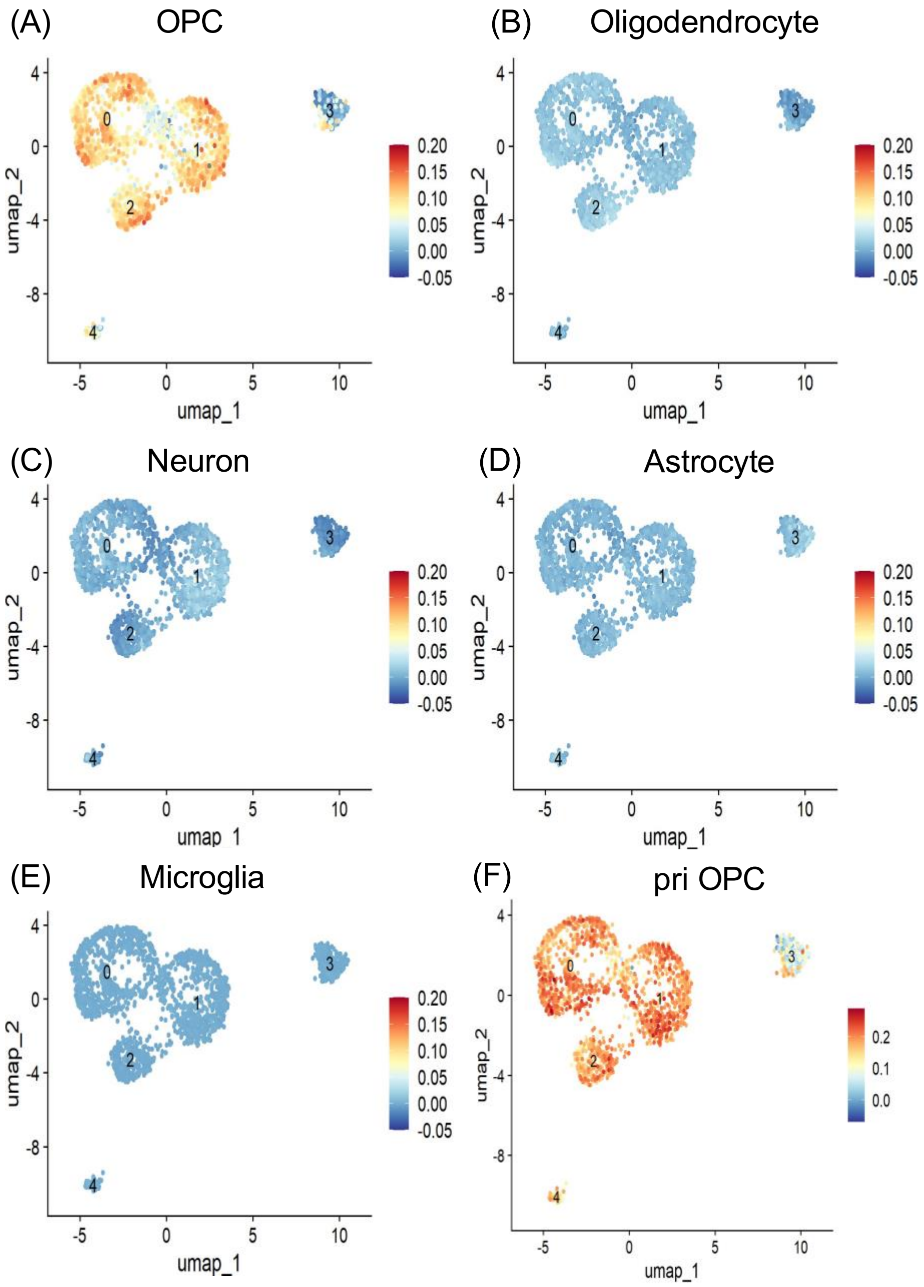

Supplemental Figure 3. Mutant BB-p53n OPCs express lineage-specific markers.

(A)-(F) UMAP plots of BB-p53n OPCs. Color-coded dots represent the relative enrichment for each cluster of the indicated cell-specific signatures, such as OPC (A), Oligodendrocyte (B), Neuron (C), Astrocyte (D), Microglia (E) and primitive OPC (F). Shades of blue represent less enrichment and shades of orange and red represent progressively higher enrichment.

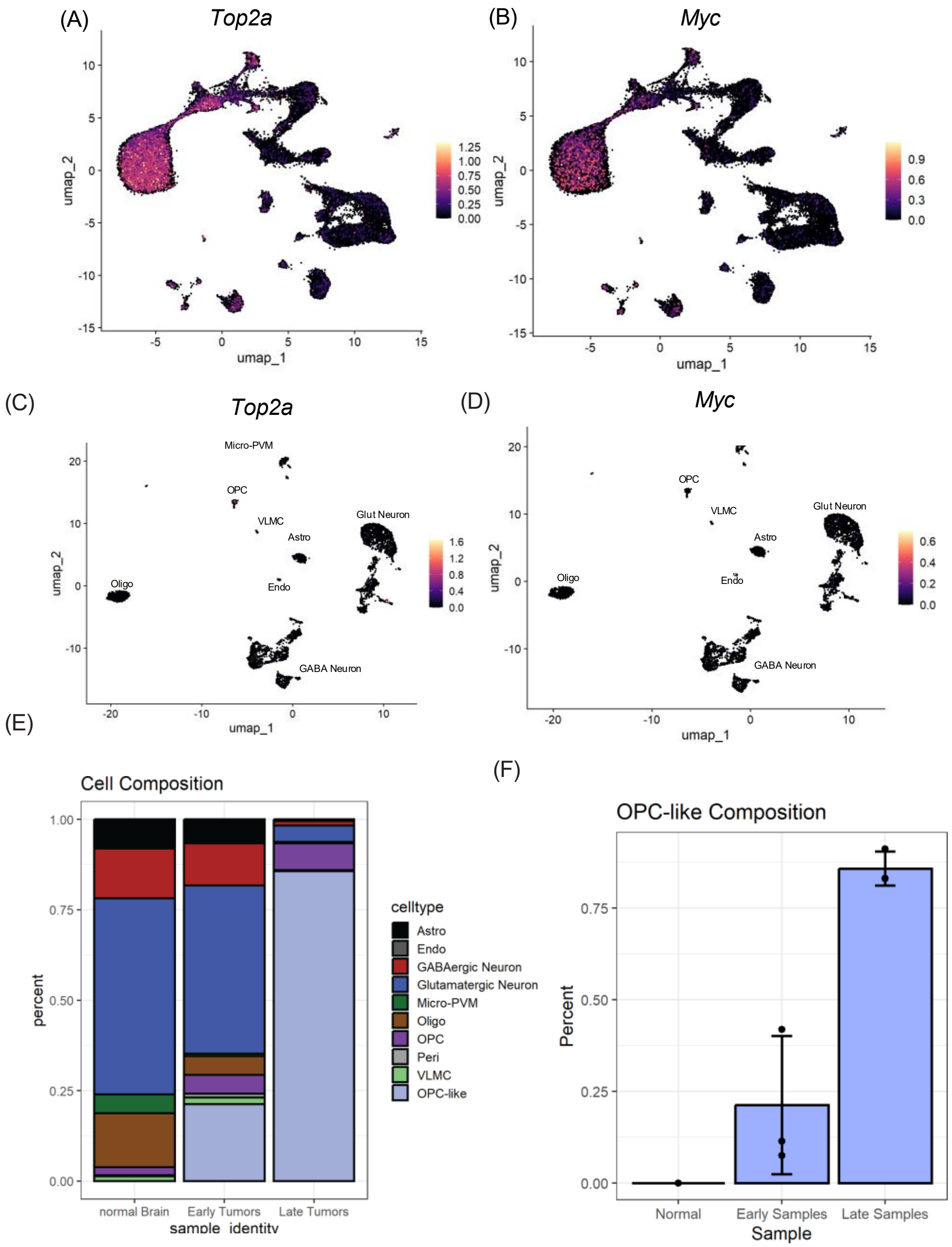

Supplemental Figure 4: The "OPC-like" clusters, characterized by *Top2*a and *Myc* expression, are not detected in the normal mouse brain dataset, and they are progressively larger in early- and late-stage tumor-bearing brain tissue samples. (A)-(B) UMAP plots of integrated early and late-stage brain datasets visualizing the cluster distribution of markers of the "OPC-like", *Top2a* and *Myc*. (C)-(D) UMAP plots of normal mouse brain datasets visualizing the cluster distribution of markers of the "OPC-like", *Top2a* and *Myc*. (E) Stacked barplots of cell type ratios in normal brain, early and late-stage samples. Note the expansion of the "OPC-like" signature cluster at the expense of other cell types. CNS cell types included are Astrocyte (Astro), Endothelial (Endo), Glutamatergic Neuron (Glut), GABAergic Neuron, Oligodendrocyte (Oligo),

Oligodendrocyte Progenitor Cell (OPC), Microglia (Micro), Pericyte (peri), and Vascular Leptomeningeal cell (VLMC). Most cell types in the late-stage samples are not visible, due to small percentages. (F) Barplot visualizing OPC-like composition by mouse between Normal, Early, and Late-stage samples. The bars visualize the mean OPC-like composition percent, the dots represent percent for each mouse analyzed and the error bars represent standard deviation.

Neftel NPC-2 GBM Signature

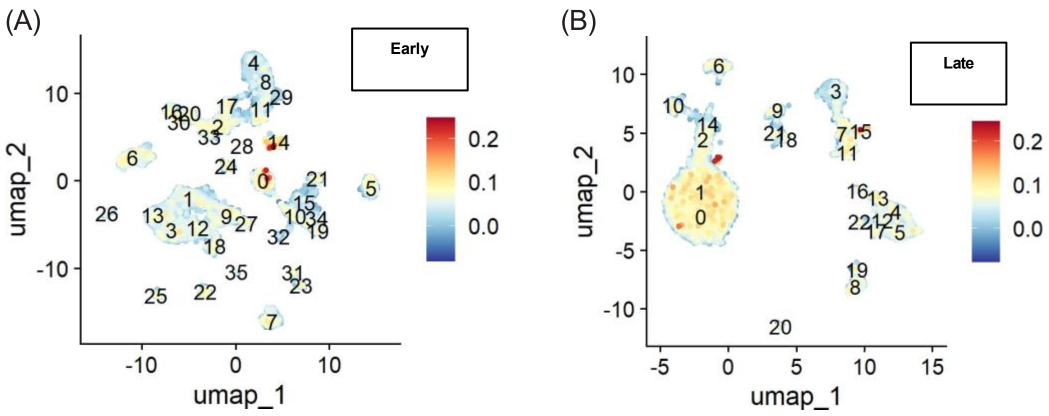

Supplemental Figure 5: Glioma model shows enrichment of human NPC GBM signatures
UMAP plots of the integrated normal brain dataset with the results from either early- (A) or late- (B) stage tumor-bearing brain tissue samples visualizing enrichment of the previously reported human glioblastoma NPC-2 signature from Neftel et al., 2019. Color gradient reflecst enrichment scores with red representing high enrichment and blue low enrichment.

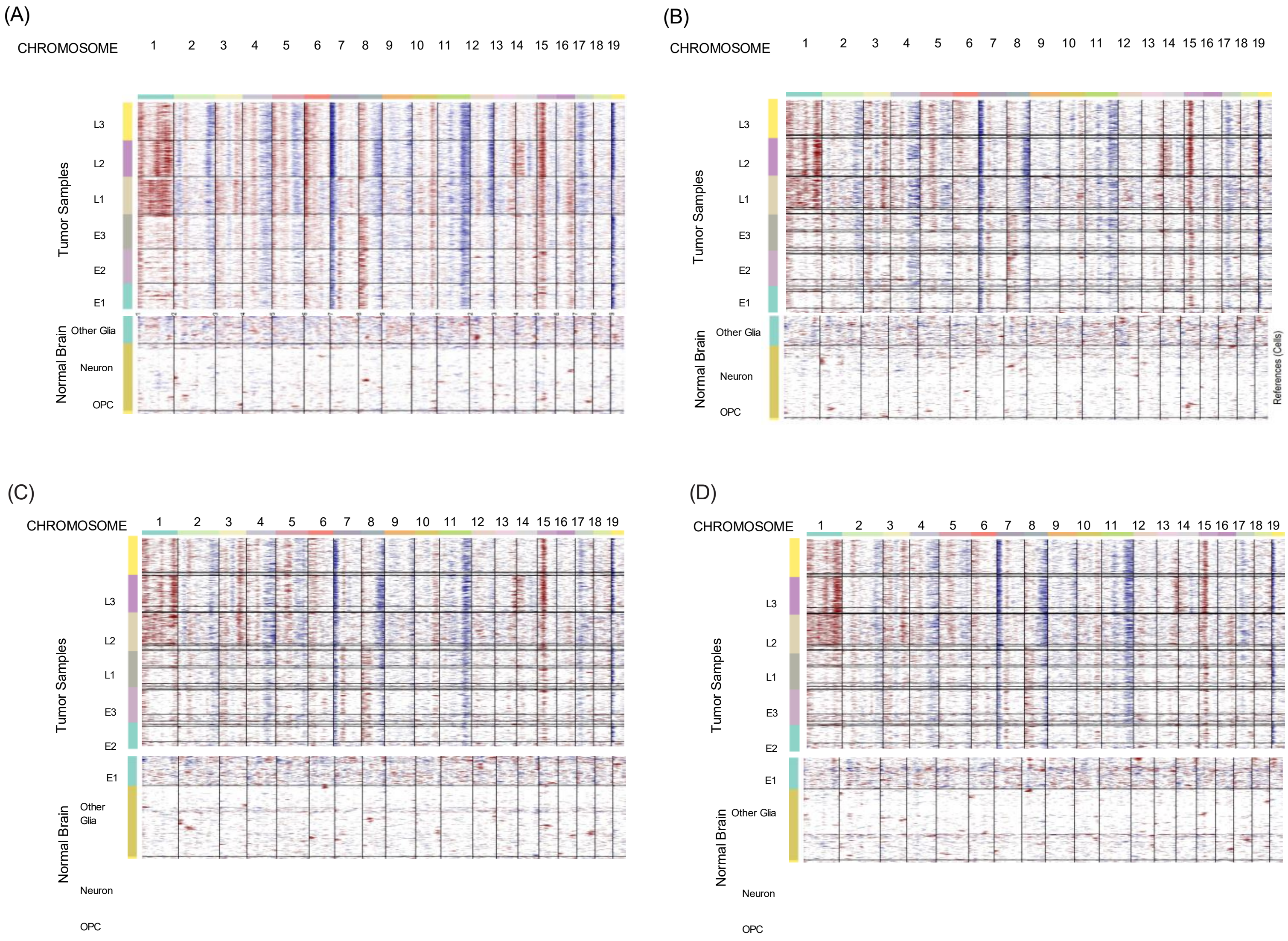

Supplemental Figure 6. Reproducibility of the InferCNV analysis.
The four panel represents the heat maps of the chromosomal distribution of inferred CNVs in four draws of randomly selected 20,000 nuclei at a time, to cover all the 80,000 nuclei analyzed from the early-and late stage tumor-bearing brain tissue samples. Each column represents the distribution along the chromosome delineated on the top by a color and a number. Each row identifies the genomic location in each of the samples from which the inferred CNV were derived. Note the three early- (E1,E2,E3) and three late- (L1,L2,L3) stage tumor bearing tissue samples. For the normal brain datasets, we ran the same analysis and separated neurons and OPC from other glial cells. Red horizontal lines represent duplications and amplifications at given chromosomal locations, blue horizontal lines represent deletion or losses. Note the progressive increase of CNVs from early to late-stage samples, especially in the gene-dense chromosome 1.

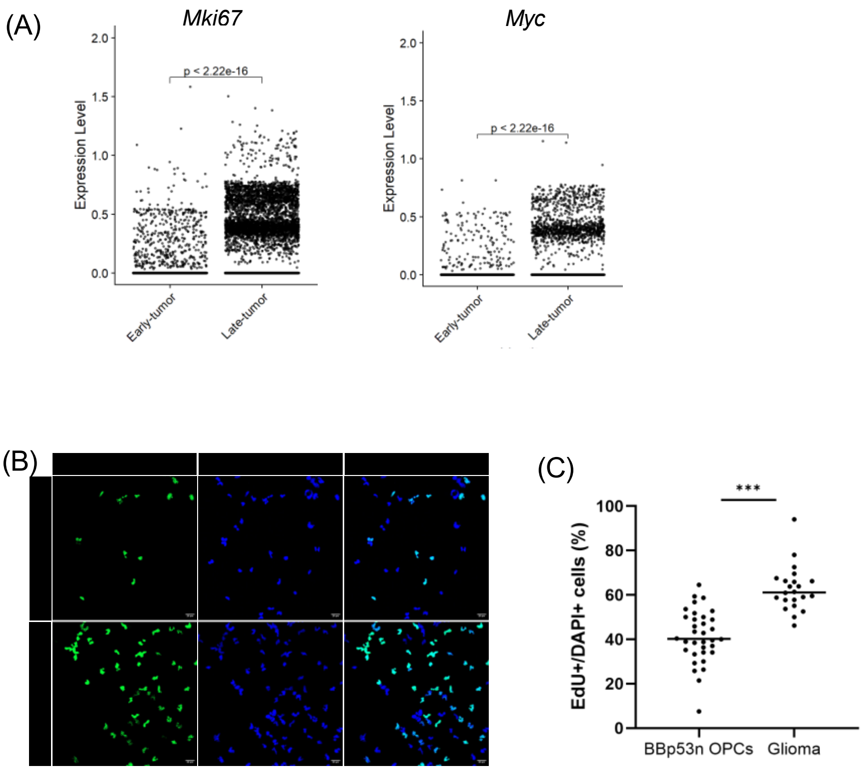

Supplemental Figure 7: Proliferative phenotype of glioma cells in vivo and in vitro. (A) Scatter plots illustrating the normalized expression levels of the cell cycle genes *Mki67* and *Myc* in early- and late-stage tumors from snRNA-seq data. *p*-value < 2.22e-16 calculated by Student's t-test. (B) Representative confocal images of EdU+ (green) glioma cells and BB-p53n OPCs. DAPI (blue) as a nuclear counterstain. Scale bar = 20um (C) Scatterplot of quantified immunofluorescence data from panel B. Each point represents the percentage of EdU+/DAPI+ cells per image. A total of 32 images were quantified for BB-p53n OPCs and 21 images for glioma cells, from eight wells for each of the biological replicates for each population. *** *p<0.001* unpaired t-test (actual value *P=0.0005*).

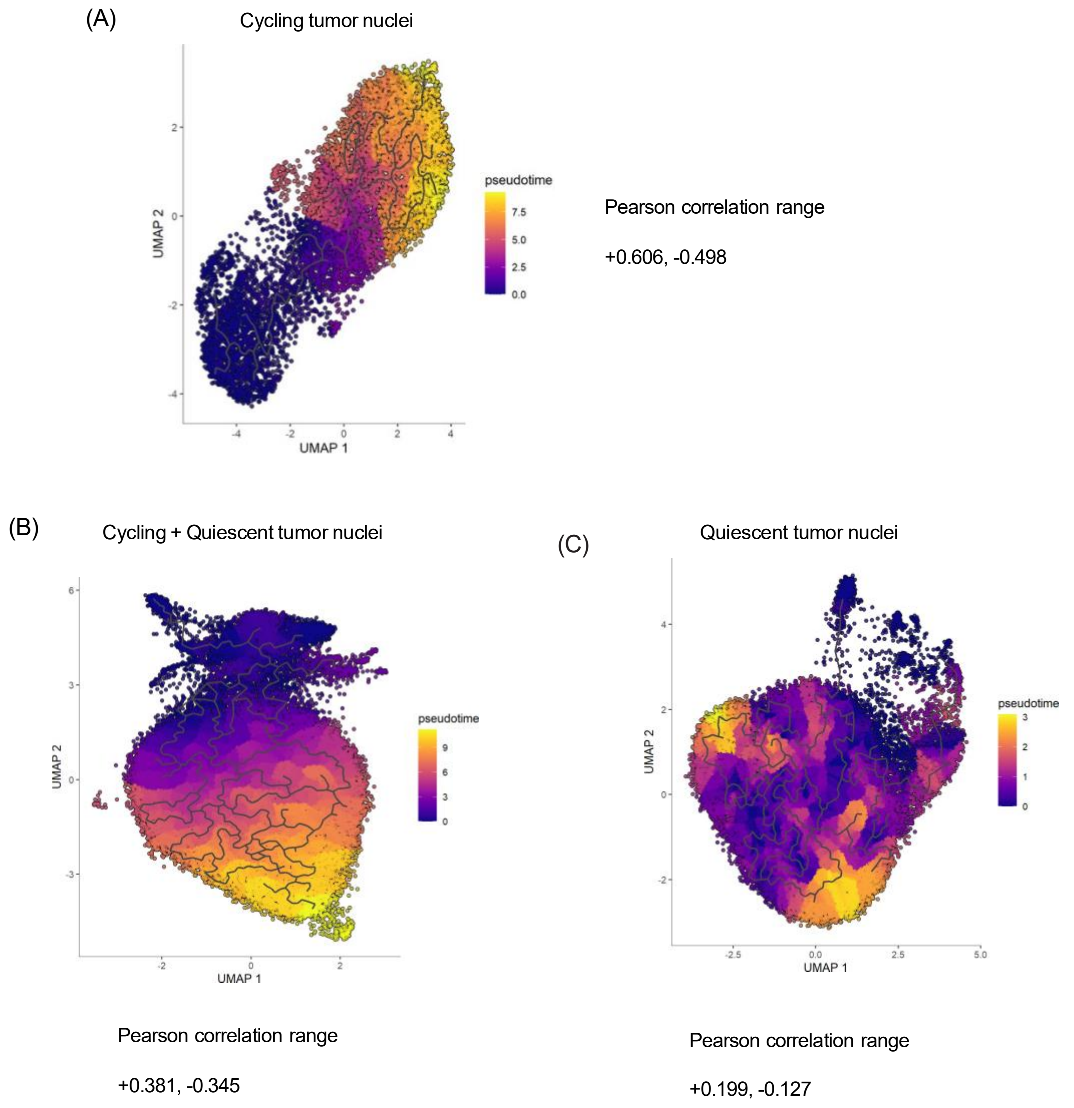

Supplemental Figure 8: Cycling tumor cells show the greatest correlation between pseudotime and gene expression.

(A)-(C) UMAP plots visualizing inferred pseudotime by monocle3, dark purple to yellow gradient, and potential branching trajectory, lines within UMAPs. (A) The most significant range of correlation was found for the pseudotime analysis of only cycling tumor cells (+0.606 to -0.498). (B) If also quiescent cells were included in the analysis, the correlation range was between +0.381 to -0.345 and (C) when only quiescent tumor cells were included, the correlation was even lower +0.199 to -0.127.

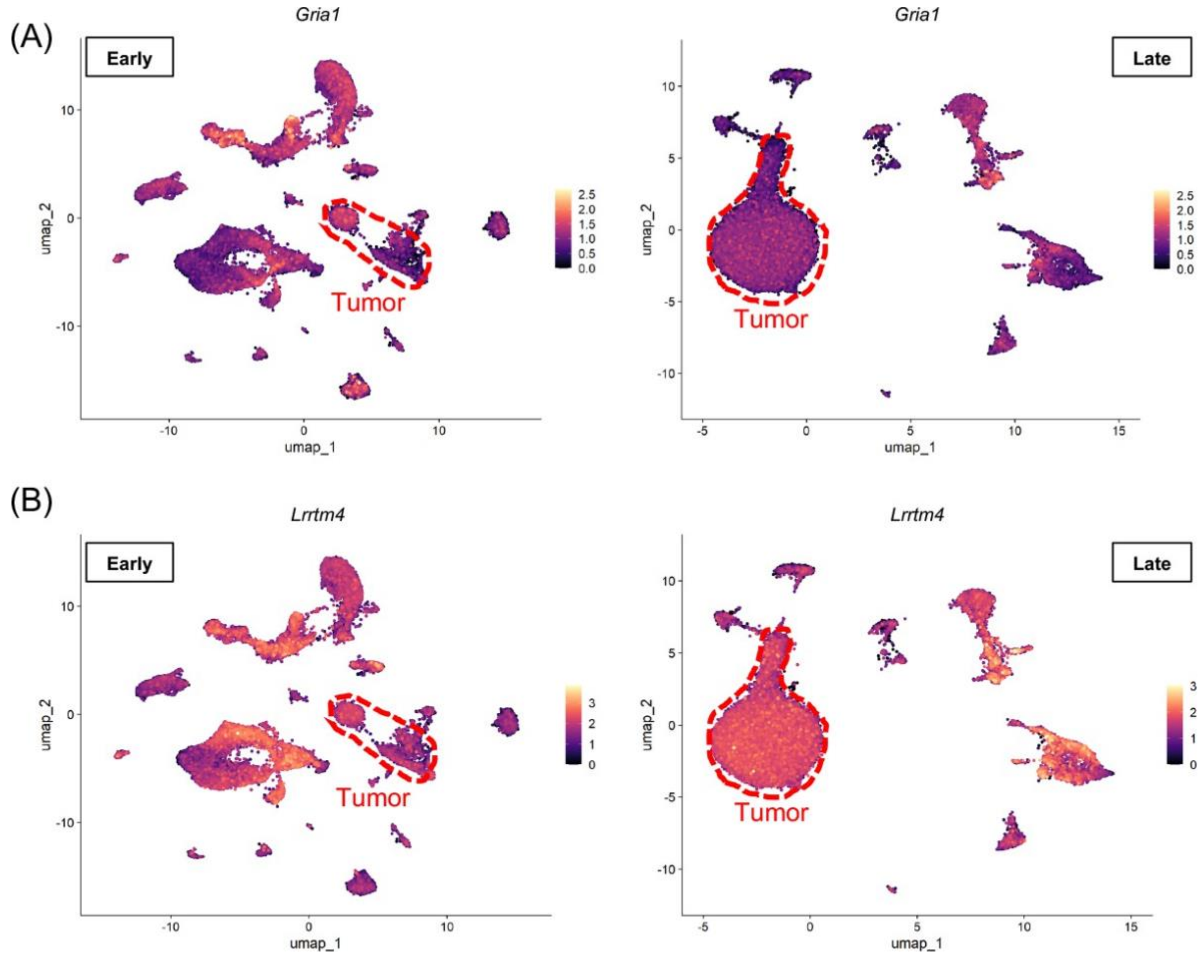

Supplemental Figure 9: Tumor cells express key post-synaptic proteins
(A) UMAP plots of integrated early and late samples integrated with normal brain visualizing gene expression levels of *Gria1*. Identified tumor cells surrounded by red dotted line. (B) UMAP plots of integrated early and late samples integrated with normal brain visualizing gene expression levels of *Lrrtm4*.

Legends for Supplemental Tables:

Supplemental Table 1. QC of the datasets.
Sample IDs are listed in the first column. Shown are the number of nuclei analyzed per sample, the mean UMI counts per cell, and the mean number of detected genes before and after filtering. Please refer also to Supplemental Figure 1.

Supplemental Table 2. The "OPC-like" gene signature.
Differentially expressed genes between "OPC-like" clusters and all the other cell types, analyzed from the integrated datasets from normal brain and from three early- and three late-stage tumor-bearing brain samples.

Supplemental Table 3. GSEA of the "OPC like" gene signature.
Results of GSEA based on the differential expression data shown in Supplemental Table 2, filtered for adjusted *p* value <=0.01.

Supplemental Table 4. Differential expression between early and late stage "tumor clusters".
Differential gene expression between the filtered out "tumor clusters" from the three early-stage and three late stage-tumor-bearing samples

Supplemental Table 5. GSEA of the early and late "tumor" clusters.
Results of GSEA based on the differential expression data shown in Supplemental Table 4, filtered for adjusted *p* value <=0.01.

Supplemental Table 6.
Pearson's correlation between gene expression and pseudo-time values
