# Supplementary material for: Single-nucleus transcriptomics identifies cell cycle and synaptic pathway dysregulation during OPC-to-glioma progression
Source: Front Cell Neurosci. 2026 Jun 30;20:1713437. doi: 10.3389/fncel.2026.1713437 (PMC13424661; doi:10.3389/fncel.2026.1713437)
Supplement: Supplementary file 2 [file Table_1.DOCX]

**Supplemental Table 1.**

|  | Before Manual Filtering | | | After Manual Filtering | | |
| --- | --- | --- | --- | --- | --- | --- |
| Sample | # of nuclei | Mean UMI / cell | Mean n genes detected | # of nuclei | Mean UMI /cell | Mean n genes detected |
| E1 | 10599 | 6226.3 | 2363.4 | 10490 | 5941.6 | 2322.5 |
| E2 | 14056 | 5098.7 | 2183.7 | 13992 | 5040.2 | 2174.2 |
| E3 | 14397 | 3722.8 | 1687.7 | 14381 | 3686.2 | 1681.8 |
| L1 | 20887 | 1634.6 | 970.8 | 14972 | 1636.1 | 971.5 |
| L2 | 16878 | 2341.1 | 1317.2 | 14985 | 2342.7 | 1318 |
| L3 | 15284 | 2485.3 | 1347.9 | 14991 | 2485.3 | 1347.9 |
| BBp53n OPCs | 10036 | 15381.4 | 4568.8 | 1954 | 15395.4 | 4774.4 |
| Normal mouse brain | 82312 | 832.4 | 454.7 | 6525 | 5610.5 | 2344.3 |

Sample IDs are listed in the first column. Shown are the number of nuclei analyzed per sample, the mean UMI counts per cell, and the mean number of detected genes before and after filtering.
